# Prospective Monitoring Reveals Dynamic Levels of T Cell Immunity to Mycobacterium Tuberculosis in HIV Infected Individuals

**DOI:** 10.1371/journal.pone.0037920

**Published:** 2012-06-07

**Authors:** Jessica E. Mitchell, Shivan Chetty, Pamla Govender, Mona Pillay, Manjeetha Jaggernath, Anne Kasmar, Thumbi Ndung’u, Paul Klenerman, Bruce D. Walker, Victoria O. Kasprowicz

**Affiliations:** 1 Ragon Institute of MGH, Massachusetts Institute of Technology (MIT) and Harvard, Harvard Medical School, Boston, Massachusetts, United States of America; 2 Kwazulu-Natal Research Institute for Tuberculosis and Human Immunodeficiency Virus (HIV) (K-RITH), Nelson R. Mandela School of Medicine, Durban, South Africa; 3 Human Immunodeficiency Virus (HIV) Pathogenesis Programme, Doris Duke Medical Research Institute, Nelson R. Mandela School of Medicine, Durban, South Africa; 4 Division of Rheumatology, Immunology and Allergy, Brigham and Women’s Hospital, Harvard Medical School, Boston, Massachusetts, United States of America; 5 Oxford Biomedical Research Centre and James Martin School for 21st Century, Nuffield Department of Medicine, University of Oxford, Oxford, United Kingdom; National Institute of Infectious Diseases, Japan

## Abstract

Monitoring of latent *Mycobacterium tuberculosis* infection may prevent disease. We tested an ESAT-6 and CFP-10-specific IFN-γ Elispot assay (RD1-Elispot) on 163 HIV-infected individuals living in a TB-endemic setting. An RD1-Elispot was performed every 3 months for a period of 3–21 months. 62% of RD1-Elispot negative individuals were positive by cultured Elispot. Fluctuations in T cell response were observed with rates of change ranging from −150 to +153 spot-forming cells (SFC)/200,000 PBMC in a 3-month period. To validate these responses we used an RD1-specific real time quantitative PCR assay for monokine-induced by IFN-γ (MIG) and IFN-γ inducible protein-10 (IP10) (MIG: r = 0.6527, p = 0.0114; IP-10: r = 0.6967, p = 0.0056; IP-10+MIG: r = 0.7055, p = 0.0048). During follow-up 30 individuals were placed on ARVs and 4 progressed to active TB. Fluctuations in SFC did not correlate with CD4 count, viral load, treatment initiation, or progression to active TB. The RD1-Elispot appears to have limited value in this setting.

## Introduction

The identification of latent tuberculosis infection (LTBI) and preventative therapy is important for TB control, especially in those most likely to progress to active tuberculosis (TB). In countries with low rates of tuberculosis those at increased risk of TB exposure are encouraged to partake of serial testing for latent Mycobacterium tuberculosis infection (LTBI) [1]. However, the identification of LTBI in individuals co-infected with HIV in high-burden TB settings is equally important due to the increased risk of progression to active TB. Isoniazid (INH) treatment of LTBI is being promoted as a major TB control strategy by the WHO [2] and accurate monitoring of LTBI status in HIV and Mycobacterium tuberculosis (MTB) co-infected individuals may aid the improved targeting of INH preventative therapy. Importantly, longitudinal monitoring of these individuals may enable timely therapy in those who have progressed, or are in the process of progressing to active TB. The diagnosis of LTBI requires the identification of an MTB-specific immunologic response. The tuberculin skin test (TST) is traditionally used to diagnose LTBI. However, its sensitivity is greatly reduced in HIV co-infected individuals due to HIV-induced cutaneous anergy resulting in false negative results [3], [4]. Serial TST testing is rarely performed.

Novel and improved diagnostics for LTBI that are predictive of the benefit of INH and/or progression to active TB are urgently required. IFN-γ release Assays (T-SPOT.TB (Elispot platform) and QuantiFERON-TB Gold (Elisa platform)) (IGRAs) have recently been introduced as a diagnostic for LTBI and are in regular use in clinical practice in the US, UK and India [5]. IGRAs are reported to perform better in an HIV infected population compared to the TST [6], [7], [8], [9], [10], [11]. However, little is currently known on the best way to interpret serial testing for LTBI using IGRAs, and this is particularly true for HIV positive populations living in highly endemic TB settings [12]–[13] [14], [15] [16], [17]. Limited data is also currently available on the predictive value of IGRAs for progression to active TB and better guidelines are clearly needed.

In contact investigations, IGRAs have been shown to better correlate with exposure to active Tuberculosis cases than TST [18], [19], [20], [21]. It has been suggested that T cell responses, as detected by IGRAs, correlate with bacterial burden [22], [23], [24], [25], [26]. In addition, serial testing using Quantiferon Gold in Tube was recently reported as a useful method for the diagnosis of new LTBI in Dutch military personnel [27]. We hypothesized that measuring an RD-1 specific immune response longitudinally by ex vivo IFN-gamma Elispot would be useful to diagnose LTBI, and potentially predict progression to active TB. Using an in-house RD1- specific IFN-gamma Elispot assay this study aimed to prospectively monitor the MTB-specific T cell response in an HIV-infected population with no signs and symptoms of active TB, and no obvious reference point in time for exposure to TB.

## Methods

### Study Participants

163 HIV chronically infected (antiretroviral therapy (ARV) naïve) individuals (CD4 T cell count range at baseline  = 5–1142 cells/ul) without signs and symptoms of active TB were recruited in Durban, South Africa. Blood samples were taken from all participants to perform a baseline assessment. All individuals had subsequent blood draws every 3 months for a period of 3–21 months. All study participants were of South African origin and presumed to have been BCG vaccinated at birth. Ethical approval was obtained from McCord Hospital, the University of KwaZulu-Natal and Massachusetts General Hospital. Written informed consent was obtained from all study participants.

**Figure 1:**
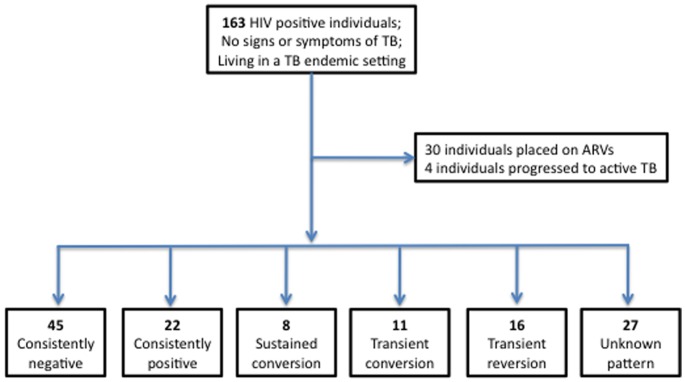
Schematic representation of study results. 163 chronically HIV infected (ARV naïve) individuals (CD4 = 5–1142 cells/ul) were recruited in Durban, South Africa. An RD1-Elispot was performed on PBMC every 3 months for a period of 3–21 months. During the period of follow-up 30 individuals were placed on ARVs and 4 individuals progressed to active TB. Of the 129 individuals who were not placed on ARVs or TB treatment during follow-up, 5 categories of Elispot kinetics were observed: 45 (35%) individuals were consistently negative at each time-point tested, 22 (17%) individuals were consistently positive at each time-point tested, 8 (6%) individuals displayed sustained conversions (three or more positive Elispots after at least one negative Elispot), 11 (9%) individuals displayed transient conversions (one or two positive Elispots between negative plates), and 16 (12%) individuals displayed transient reversions (one or two negative Elispots between positive plates). For 27 (21%) individuals the category is unknown due to either a lack of interpretable results (e.g. SK 179 had indeterminate Elispots at all 3 time points tested), or the absence of either pre-baseline samples or further follow-up to determine if observed reversions and conversions are transient or sustained.

### Elispot Assays

All participants were tested for RD1 (ESAT-6 and CFP-10)-reactivity using an ex vivo IFN-γ Elispot assay (RD1-Elispot) on fresh PBMC (method described in [28]). ESAT-6 and CFP-10 peptide pools were added at a final peptide concentration of 8 ug/ml. Phytohemagglutinin (PHA) (10 µg/ml) was used as a positive control. PBMC were plated at 200,000 cells/well and incubated overnight at 37°C and 5% CO2. All stimulations were performed in triplicate and reported as the average. RD1- specific responses were considered positive if the number of spots per well was four SFC more than negative control wells and this number was at least twice that in negative control wells. This pre-defined cut-off point translates into a detection threshold of 20 peptide-specific T cells per million PBMC [6], [28] [11]. An Elispot was considered positive if a positive result was obtained by stimulation with ESAT-6, CFP-10 or both. An indeterminate result was one that had a positive control less than 20 SFC per 200,000 PBMC or a negative control greater than 10 SFC per 200,000 PBMC [29]. A conversion was defined as a negative Elispot followed by a positive Elispot, while a reversion was considered the opposite [30].

**Figure 2:**
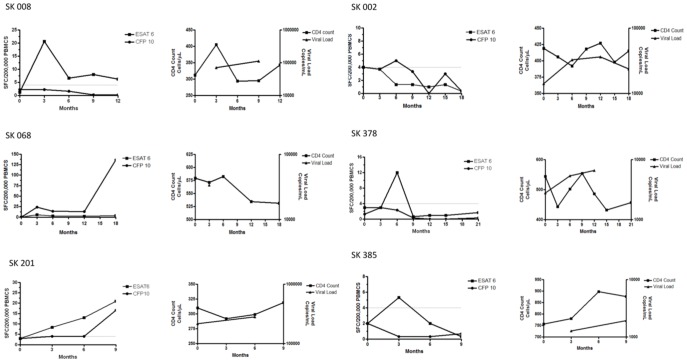
Longitudinal MTB-specific T cell profiles and correlation with CD4 count and HIV viral load. Longitudinal Elispot data from individuals who were not on ARVs for the entire period of follow-up. Figure 2A: Examples of responses from 3 individuals who were ex vivo RD1-Elispot positive at all time-points during follow-up. Figure 2B: Examples of responses from 3 individuals who displayed an RD1-Elispot conversion that was sustained during follow-up and examples of responses from 3 individuals who displayed a transient RD1-Elispot conversion during follow-up. Figure 2C: Examples of responses from 3 individuals who displayed a transient RD1-Elispot reversion during follow-up. Left-hand panel =  Elispot data, right-hand panel  =  CD4 counts and viral loads.

### Cultured Elispot

4×10^6^ PBMCs were cultured with either ESAT-6 or CFP-10 peptide pools at a final peptide concentration of 8 ug/ml, for one hour at 37°C and 5% CO_2_. The cells were then plated in R10 media at a concentration of 10^6^/2 mL and cultured for 2 weeks at 37°C and 5% CO_2_. On the third day of culture, 1 mL of the culture media was replaced with 1 mL R10–50 Media (R10 media supplemented with 100 units/ul IL-2); the media was refreshed in this manner every 3 three days. After two weeks, the cells were washed three times with R10 media, re-plated and allowed to rest overnight in the incubator. The cells were then washed twice with R10, counted and a routine RD1-Elispot performed.

**Figure 3:**
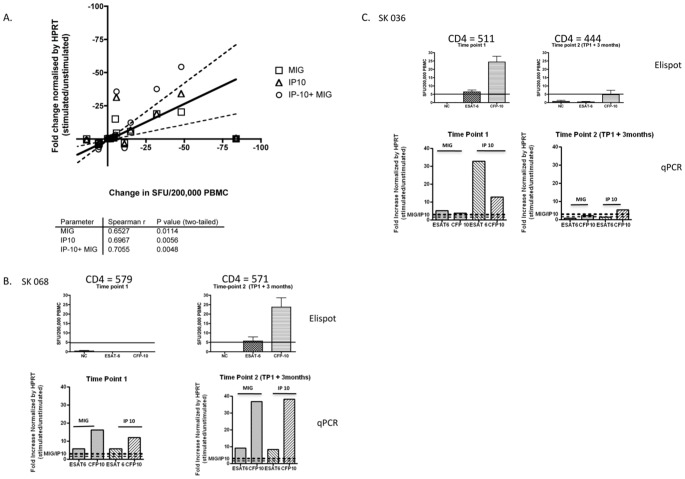
Longitudinal comparison of the ex vivo RD1- Elispot and the RD1-qPCR assay. A highly sensitive assay of RD1-specific IFN-γ production using real time quantitative PCR (qPCR) for two reporters - monokine-induced by IFN-γ (MIG) and the IFN-γ inducible protein-10 (IP10) was compared longitudinally with the ex vivo RD1-Elispot. Figure 3A: A head-to–head comparison was performed on 14 individuals on samples from 2 time-points 3 months apart. A significant correlation was observed between fold change on the RD1 qPCR and change in SFC using the RD1-Elispot. Figure 3B: Data from SK068. Figure 3C: Data from SK036. Top panel displays RD1-Elispot data and bottom-panel displays RD1-qPCR data.

### RD1-specific qPCR Assay

We used a highly sensitive assay of ESAT-6 and CFP-10-specific IFN-γ production using real time quantitative PCR (qPCR) for two reporters - monokine-induced by IFN-γ (MIG) and the IFN-γ inducible protein-10 (IP10) (RD1-qPCR). This assay was performed as described in Kasprowicz et al [31]. In brief, PBMCs (10^6^ cells/condition) were stimulated with 10 ng/ml recombinant IFN-γ (positive control), 25 µl of R10 media (null), or ESAT-6 and CFP-10 peptide pools (final peptide concentration of 8 µg/ml) at 37° and 5% CO_2_ for 16 hours. mRNA extraction was performed using an RNeasy mini kit (Qiagen); extracted cellular mRNA was reverse transcribed by RT-PCR using iScript cDNA synthesis kit (BIORad) using 10 µl of cellular mRNA; and qPCR for MIG, IP10 and HPRT was performed.

## Results

### Cross-sectional Assessment of RD1 Reactivity by Ex-vivo Elispot Suggests that 40% of the HIV+ Individuals have Latent MTB Infection

Of the 163 baseline Elispots performed, 8 (4.9%) were indeterminate (6 due to high assay background and 2 due to failed positive control) (Table S1). In Elispot positive individuals, the ESAT-6-specific responses ranged from 4–98 spot-forming cells (SFC) (mean of 19), while the CFP-10 responses ranged from 4–87 SFC (mean of 20). 66 (40%) of the 163 individuals with evaluable results were found to be ex-vivo IFN-gamma Elispot positive (indicating suspected LTBI) (Table S1). Interestingly, 18/66 (27%) of positive patients had RD-1 specific responses within 5 SFC of the cut-off. No significant correlation between CFP-10 SFU and CD4 count (r = −0.07185, P = 0.3621) and ESAT-6 SFU and CD4 count (r = −0.05382, P = 0.4950) was found (data not shown).

### Longitudinal Analysis of RD1-specific T Cell Responses by Elispot Reveals a High Level of T Cell Fluctuation

All study participants were tested by ex vivo RD1-Elispot at baseline and 3 months later. 157 individuals had subsequent blood draws every 3 months for a period of up to 21 months (Table S1). Interestingly, of the 737 Elispots displayed in the data set in Table S1, 66 (9%) indeterminate assays were reported. Of the 163 participants listed, 30 individuals were placed on ARV treatment during follow-up, 2 were placed on TB treatment, and 2 were placed on both ARV and TB treatment. Of the 129 individuals who were not placed on ARVs or TB treatment during follow-up, 5 categories of Elispot kinetics were observed: 45 (35%) individuals were consistently negative at each time-point tested, 22 (17%) individuals were consistently positive at each time-point tested, 8 (6%) individuals displayed sustained conversions (three or more positive Elispots after at least one negative Elispot), 11 (9%) individuals displayed transient conversions (one or two positive Elispots between negative plates), and 16 (12%) individuals displayed transient reversions (one or two negative Elispots between positive plates) (Figure 1). For 27 (21%) individuals the category was unknown due to either a lack of interpretable results, or the absence of either pre-baseline samples or further follow-up to determine if observed reversions and conversions were transient or sustained. Figure 2 A, B, C displays examples of 4 of the main categories of Elispot kinetics.

**Figure 4:**
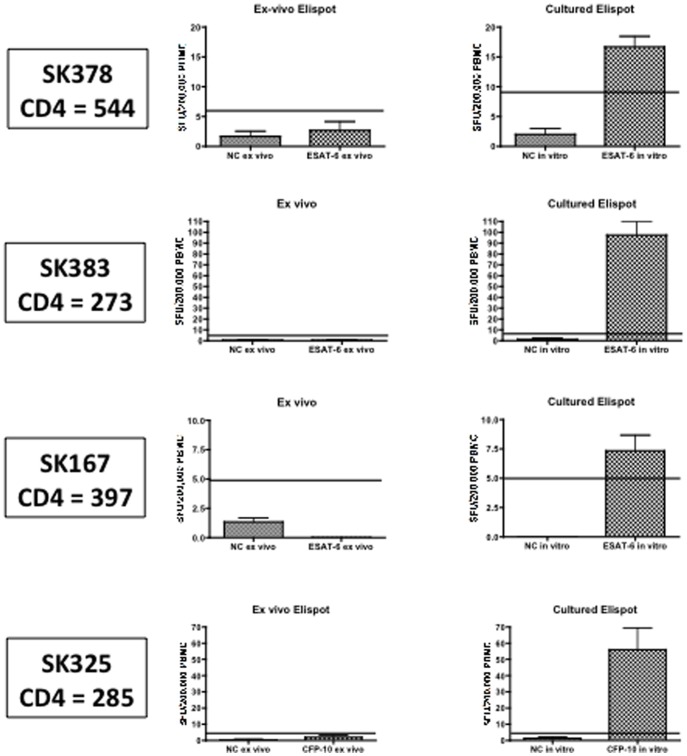
In- vitro culture reveals RD1-specific T cell responses. Left-hand panel displays ex vivo IFN-gamma Elispot data from 4 individuals (SK378, SK383, SK167, and SK325) who were ex vivo Elispot negative at 2 time-points 3 months apart. Right-hand panel displays data from the same individuals following a cultured Elispot (14 day in vitro culture of PBMC with the ESAT-6 or CFP-10 peptides followed by IFN-gamma Elispot). All individuals had CD4 counts above 250.

The majority of individuals (52%) maintained assay status throughout the follow-up. However, despite this, significant fluctuations in SFC were observed. Of those subjects that were consistently Elispot positive, a range of T-cell fluctuations were observed over time. For example, SK036 showed a response of 24 SFC to CFP-10, which decreased after 3 months to 4 SFC, but then increased again after a further 3 months to 83 SFC, and subsequently decreased to 34 SFC (figure 2A). This is a fluctuation of approximately 50 SFC every 3 months. We were unable to quantify all responses due to particular wells appearing completely black (‘black-out’), most likely due to an extreme rise in RD1-specific responses. For those who were consistently Elispot positive, the maximum change in SFC in a 3-month period was −150 and +153 SFC for ESAT-6-specific responses and −45 and +45 SFC for CFP-10 specific responses. Overall, the change in SFC in a 3-month time-period ranged from −150 to +153 SFU for ESAT-6-specific responses and −88 and +123 SFC for CFP-10 specific responses.

Of the transient conversions, 11 (55%) had positive responses that were within 5 SFC of the assay cut-off, indicating that these borderline responses may be false-positives (highlighting the obvious but important association between cut-off selection and assay status). Interestingly, the change in SFC between transition time-points (i.e. the two time-points over which an Elispot status change occurs) is statistically different when comparing individuals displaying transient conversions (mean  = 8 SFC) and those displaying sustained conversions (mean  = 17 SFC) (p = 0.0071, Mann-Whitney t-test). However, if you remove the 6 individuals that lie within 5 SFC of the cut-off, the difference no longer exists, indicating that a sub-set of individuals displaying transient conversions have a change in SFC in the same range as those with sustained conversions. The change in SFC between transition time-points for individuals displaying transient reversions ranged from −3 to −37 (mean  = −13 SFC). Almost 35% of individuals in our cohort were consistently negative at all time-points despite living in a TB endemic setting. The observation of borderline positive results highlights the potential for MTB-specific responses to occur below the selected assay cut-off and even below the threshold of the ex vivo RD1-Elispot assay.

**Figure 5:**
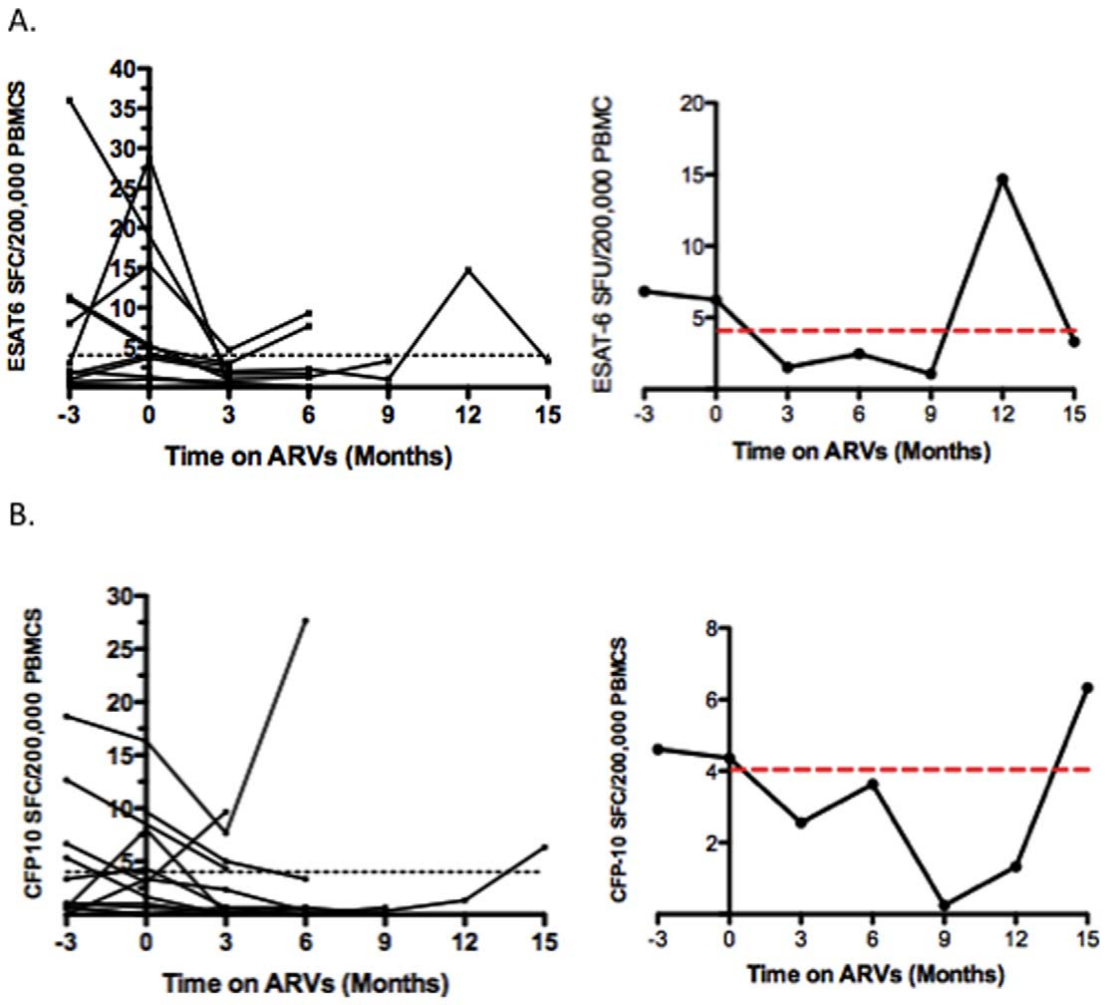
Impact of ARVS on the RD1-specific T cell kinetic profile. Figure 5 A–B: Summary of longitudinal data from 11 individuals who were placed on ARVs and had a consistent cut-off at all time-points tested. Figure 5A: Summary of ESAT-6-specific responses. Figure 5B: Summary of CFP-10-specific responses. Left-hand panel =  data from all 11 individuals. Right-hand panel =  Mean response plotted. The mean ESAT-6 spot-forming cells (SFC) at 3 months prior to ARV initiation was 7, and that for CFP-10 was 5 SFC. These responses declined to 2 SFC for ESAT-6 specific responses and 3 SFC for CFP-10 specific responses. Overall the mean response declined after the initiation of ARV therapy, however this did not reach statistical significance (ESAT-6: p = −0.1133, CFP-10: p = 0.1109 (paired t-test)).

### RD1-specific Molecular Assay Provides Independent Support for the Observed RD1-Elispot Fluctuations

The RD1-qPCR has recently been developed, validated and applied to the detection of MTB-specific responses [31]. To assess whether the variability in Elispot responses could be validated using a separate assay platform - and are therefore biologically valid - the *ex vivo* Elispot and the qPCR assays were compared longitudinally. A head-to-head comparison was performed on 14 individuals on samples from 2 time-points 3 months apart. A clear quantitative correlation was observed between the two assays over time (figure 3A). Figures 3B and C show examples from two patients, SK068 and SK036. SK068 shows an Elispot assay conversion. However, this conversion was not observed when performing the qPCR assay, which was positive at both time-points, although changing quantitatively, consistent with the enhanced sensitivity of the qPCR [31].

**Figure 6:**
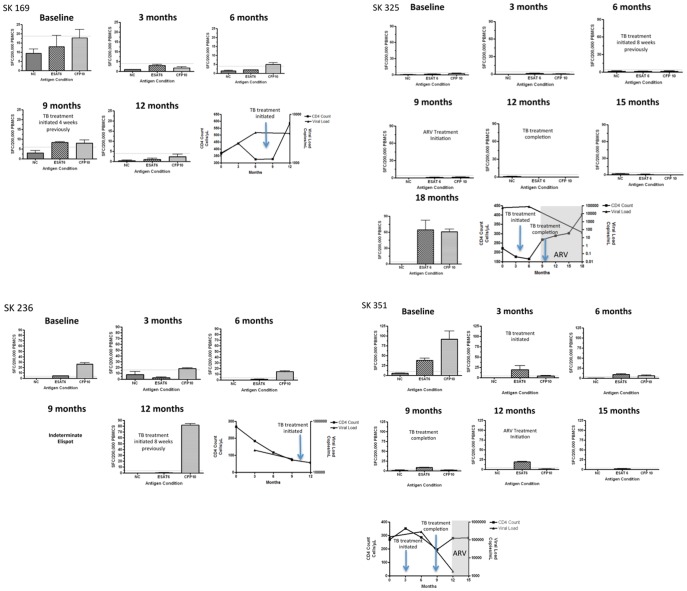
Prospective monitoring of RD1- specific IFN-gamma Elispot responses does not predict progression to active TB. Longitudinal Elispots are shown for SK 169, SK 236, SK325 and SK 351 all of whom progressed to active TB during the follow-up period and 2 of which (SK 325 and SK 351) were also subsequently placed on ARVS. TB Treatment was initiated shortly after diagnosis of active TB. Longitudinal CD4 and viral load data is also shown for all 4 individuals.

### In vitro-culture Using RD1 Peptide Pools Reveals MTB-specific Responses that are Below the Threshold of Detection of the RD1-Elispot

Our results above indicate that many MTB-specific T cell populations may lie close to (but either side of) the ex vivo RD1-Elispot cut-off. To address whether sub-threshold responses existed we performed Cultured RD1-Elispots to assess the proportion of individuals with MTB specific memory responses which were not detected in the ex vivo RD1-Elispot assay [32], [33]. 13 (62%) out of 21 individuals (who were negative by ex vivo RD1- Elispot) were positive by cultured Elispot (responses ranging from 30–475 SFC/million PBMC) (data not shown and figure 4). These data indicate that MTB-specific responses may exist in a large fraction of individuals who are RD1-Elispot negative.

### RD1-specific T Cell Frequencies do not Correlate with Stage of HIV Disease Progression or ARV Status

Changes in CD4 count were not observed to correlate with changes in SFC. 32 individuals were placed on ARV therapy during the period of follow-up (due to a low CD4 count, to prevent mother-to-child transmission (PMTCT) of the virus, or as a result of diagnosis of TB). In all cases ARVs did not have a clear and consistent impact on the RD1-specific response (data not shown). Figures 5 A and B displays the summary data of 11 individuals who were placed on ARVs and had a consistent cut-off for all time-points tested. Overall the mean response declined after the initiation of ARV therapy, however this did not reach statistical significance (ESAT-6: p = −0.1133, CFP-10: p = 0.1109 (paired t-test)).

### Prospective Monitoring of RD1- specific T Cells Responses is not Able to Predict Progression to Active TB

Four individuals (SK 169, SK 236, SK 325 and SK 351) progressed to active TB during the period of follow-up (figure 6). All four did not show a striking change in RD1-specific responses upon progression to active TB. However, for SK351 the RD1-specific T cell response decreased slightly as the individual progressed to active TB (37 SFC to 19 SFC for ESAT-6, and 91 SFC to 4 SFC for CFP-10). TB treatment initiation was also not associated with striking changes in SFC. The exception to this was SK236 were TB treatment was associated with a marked increase in the CFP-10- specific response (14 SFC to 81 SFC).

## Discussion

Knowledge of the kinetics of IGRAs and their significance is limited and there is no consensus on the clinical utility of serial testing. Unlike previous studies, here we prospectively monitored MTB-specific T cell using an RD1-Elispot in an HIV-infected population living in a highly endemic TB setting, with no obvious reference point in time for exposure to TB [34], [35] [14], [15] [16], [17]. The province of KwaZulu-Natal has an estimated 1.2 million HIV positive individuals, an antenatal HIV prevalence of 37.4% and a TB notification rate of 1094/100,000 population [36], [37], [38].

We observed 5 main categories of Elispot kinetics: persistently positive, persistently negative, sustained conversions, transient conversions, and transient reversions. A significant proportion of the observed reversions and conversions in our study had borderline responses raising the issue of whether they result in non-specific variation in laboratory procedures, or whether they are a result of a true biological change [39], [40]. Elispot reversions have been reported as more common with low or moderately positive results [27]. Two recent systemic reviews on reproducibility and serial testing of health care workers both highlight that borderline conversions and reversions do occur suggesting that a simplistic dichotomous cut-off is too simplistic for interpretation of serial IGRAs [13], [40], [41]. Veerapathran et al recommend that a true conversion requires a change from a negative to a positive result and a minimum 30% increase in the baseline IFN-gamma response [42]. It is clear that defining appropriate cut-offs and criteria for assay conversions and reversions is absolutely critical to ensure that assay interpretation is both accurate and significant [39], [40], [42].

In this study we found that responses frequently lie close to the level of detection of the RD1-Elispot platform, indicating that the selected standard assay cut-off may misrepresent the level of LTBI (indicated in this cohort at 40%). The reported prevalence of LTBI in HIV infected populations ranges from 43% in a cohort from Zambia (determined by Elispot) to 89% in a cohort of South African minors (determined by TST) [4], [6]. Importantly, the RD1-qPCR is able to identify RD1-specific responses in individuals who are RD1-Elispot negative, and the cultured Elispot is able to amplify RD-1 specific responses in 62% of individuals who are repeatedly RD1-Elispot negative. It has been suggested that ex vivo IGRAs detect recent MTB infection i.e. recently activated lymphocytes (effector memory responses) and not more remote MTB infection (central memory responses), which is most likely detected by the cultured Elispot [43], [44] [44]–[45]. The RD1-Elispot and the RD1-qPCR assays appear to measure similar readouts (potentially effector memory responses targeting viable bacilli) although the qPCR assay appears more sensitive [31]. However, the RD1-Elispot is clearly able to detect MTB-specific responses of a certain magnitude and, in the absence of signs and symptoms of active TB, this response is presumably indicative of LTBI. To conclusively prove the absence of active TB in HIV positive individuals, sputum samples should be taken for AFB and culture [46]. Overall, our data suggest that MTB-specific responses lie on a spectrum, which is extended by cultured Elispot. In the absence of a gold standard for LTBI it is difficult to understand the significance of immunological sensitization to TB.

Our results indicate that it is difficult to interpret the significance of a negative RD1-Elispot result in HIV- positive individuals living in a highly TB endemic setting. It may indicate that an individual’s immune system has never encountered Mycobacterium tuberculosis, that the individual has cleared the bacterium, that the bacterium is stimulating a low-level RD1-specific response, or alternatively that the bacterium is in a dormant state. Transient reversions (and conversions) may be attributable to exposure to antigen and result as a function of the MTB life-style e.g. ESAT-6 and CFP-10 may be intermittently secreted [12]. Interestingly, 8 individuals displayed sustained conversions and the change in SFU between transition time-points was similar to a sub-set of individuals with transient conversions. A sustained conversion may indicate recent infection or bacterial reactivation. Large fluctuations in IFN-gamma responses were observed even in individuals who were persistently positive at all time-points tested. As we observed a clear quantitative correlation between the RD1-Elispot assay and the RD1-qPCR assay, this provides support that the observed MTB-specific immune fluctuations are real and not an artefact of assay performance and procedures. It is impossible to assess if any of these individuals were re-exposed or whether the fluctuations are representative of natural host fluctuations, or host-pathogen interactions. Interestingly, we did not observe a correlation between parameters of HIV disease progression (CD4 count and viral load) and change in SFC, indicating that HIV disease status did not impact significantly on the observed fluctuations in T cell response [16], [17]. In our study, ARVs were not associated with a change in RD1-Elispot status.

During the period of follow-up, 4 individuals were diagnosed with active TB and placed on TB treatment. Progression to active TB and TB treatment initiation was not associated with any observable pattern of change in RD1 SFC, nor change in RD1-assay status. Our results highlight the major issue that a key obstacle to wider treatment of LTBI is the predictive value of the currently available diagnostic tests (reviewed in Kasprowicz et al. In press Journal of Infectious Diseases). A limitation of our study is the low number of individuals that progressed to active TB. A further point to consider is that we used a generic version of the commercially available T-SPOT.TB (Elispot IGRA platform).

In conclusion, here we prospectively monitor MTB-specific responses in individuals living in a highly TB endemic setting using the RD1-Elispot. In contrast to previous studies, our study focused on a HIV positive population with no obvious reference point in time for exposure to TB. Our results highlight that a negative/positive cut-off is too simplistic for interpretation of serial RD1-Elispots [39], [40], [42]. While the RD1- Elispot is able to detect MTB-specific responses (and presumed LTBI) our data suggest that these responses lie on a spectrum, which is extended by cultured Elispot. It is therefore difficult to interpret the significance of a negative RD1-Elispot result in this setting. Large fluctuations in IFN-gamma responses were observed, even in individuals who were persistently positive at all time-points tested, and were validated by the RD1-qPCR. HIV disease status did not impact significantly on the observed fluctuations in T cell response. We find that the RD1-Elispot fails to clearly detect new MTB infection or predict progression to active disease. Thus, the RD1-Elispot, as currently used, appears to have limited value in following HIV positive individuals living in an area endemic for TB. More studies need to be performed in order to understand the clinical and biological significance of fluctuations in MTB-specific T cell responses [47] [48], [49], [50]. Regardless, the RD1-Elispot may still be a useful research tool to identify those with LTBI and facilitate immunological investigation of novel TB biomarkers in HIV infected individuals.

## Supporting Information

Table S1 **Longitudinal Elispot data for 163 study participants– BO indicates a ‘black out’ (Elispot well is purple and Spot Forming Cells can’t be individually counted).**
(DOC)
