# Supplementary material for: Prospective Monitoring Reveals Dynamic Levels of T Cell Immunity to Mycobacterium Tuberculosis in HIV Infected Individuals
Source: PLoS One. 2012 Jun 7;7(6):e37920. doi: 10.1371/journal.pone.0037920 (PMC3369919; doi:10.1371/journal.pone.0037920)
Supplement: Table S1 — Longitudinal Elispot data for 163 study participants– BO indicates a ‘black out’ (Elispot well is purple and Spot Forming Cells can’t be individually counted). (DOC) [file pone.0037920.s001.doc]

|  | **PID** | **Months follow-up** | **Ave ESAT 6** | **Ave**  **CFP 10** | **Ave**  **NC** | **Elispot Result** | **CD4 Count** | **Viral Load** | **Timepts**  **on ARV TX** | **Time points on TB Treatment** |
| --- | --- | --- | --- | --- | --- | --- | --- | --- | --- | --- |
|  |  |  |  |  |  |  |  |  |  |  |
| **1** | SK 002 | Baseline | 4.00 | 4.00 | 1.33 | neg | 419 | 14020 |  |  |
|  | SK 002 | 3 | 3.67 | 3.67 | 0.67 | neg | 406 |  |  |  |
|  | SK 002 | 6 | 1.33 | 5.00 | 0.00 | pos (CFP10) | 392 | 32656 |  |  |
|  | SK 002 | 9 | 1.33 | 3.33 | 0.67 | neg | 418 |  |  |  |
|  | SK 002 | 12 | 1.00 | 0.00 | 0.33 | neg | 427 | 35976 |  |  |
|  | SK 002 | 15 | 1.33 | 3.00 | 2.00 | neg | 398 |  |  |  |
|  | SK 002 | 21 | 0.33 | 0.33 | 0.00 | neg | 415 | 23530 |  |  |
|  |  |  |  |  |  |  |  |  |  |  |
| **2** | SK 008 | Baseline | 1.33 | 2.33 | 1.00 | neg | 312 |  |  |  |
|  | SK 008 | 3 | 20.67 | 2.33 | 0.33 | pos (ESAT6) | 405 | 70793 |  |  |
|  | SK 008 | 6 | 6.67 | 1.67 | 0.00 | pos (ESAT6) | 294 |  |  |  |
|  | SK 008 | 9 | 8.00 | 0.33 | 0.00 | pos (ESAT6) | 295 | 113721 |  |  |
|  | SK 008 | 12 | 6.33 | 0.33 | 0.00 | pos (ESAT6) | 343 |  |  |  |
|  |  |  |  |  |  |  |  |  |  |  |
| **3** | SK 009 | Baseline | 2.67 | 0.67 | 1.00 | neg | 309 | 7549 |  |  |
|  | SK 009 | 3 | 0.67 | 0.67 | 0.33 | neg | 355 |  |  |  |
|  | SK 009 | 6 | 13.00 | 4.00 | 2.33 | pos (ESAT6) | 499 | 11933 |  |  |
|  | SK 009 | 9 | 3.33 | 3.00 | 0.67 | neg | 443 |  |  |  |
|  | SK 009 | 12 | 1.00 | 1.00 | 0.00 | neg | 407 | 13702 |  |  |
|  | SK 009 | 15 | 2.33 | 0.67 | 0.00 | neg | 329 | 15107 |  |  |
|  | SK 009 | 21 | 4.00 | 4.00 | 2.00 | neg | 350 |  |  |  |
|  |  |  |  |  |  |  |  |  |  |  |
| **4** | SK 010 | Baseline | 2.00 | 2.33 | 0.67 | neg | 218 | 5822 |  |  |
|  | SK 010 | 3 | 50.33 | 43.00 | 71.67 | INDETERMINATE | 307 |  |  |  |
|  | SK 010 | 6 | 0.00 | 0.67 | 0.00 | neg | 258 | 6852 |  |  |
|  | SK 010 | 9 | 0.33 | 0.33 | 0.00 | neg | 279 |  |  |  |
|  | SK 010 | 12 | 0.00 | 0.00 | 0.67 | neg | 259 | 8805 |  |  |
|  | SK 010 | 15 | 0.00 | 0.00 | 0.00 | INDETERMINATE | 258 |  |  |  |
|  |  |  |  |  |  |  |  |  |  |  |
| **5** | SK 011 | Baseline | 1.33 | 3.33 | 2.33 | neg | 308 |  |  |  |
|  | SK 011 | 3 | 5.00 | 3.00 | 0.67 | pos (ESAT6) | 538 | 1280 | Yes |  |
|  | SK 011 | 6 | 1.67 | 1.67 | 0.33 | neg | 702 |  | Yes |  |
|  | SK 011 | 9 | 1.00 | 0.67 | 0.00 | neg | 461.16 | 702 | Yes |  |
|  | SK 011 | 12 | 104.67 | 62.33 | 0.00 | pos (ESAT6/CFP10) | 217 |  | Yes |  |
|  |  |  |  |  |  |  |  |  |  |  |
| **6** | SK 014 | Baseline | 1.00 | 0.00 | 0.33 | neg | 550 | 1358 |  |  |
|  | SK 014 | 3 | 1.00 | 1.67 | 0.00 | neg | 503 |  |  |  |
|  | SK 014 | 6 | 1.67 | 2.00 | 0.00 | neg | 495 | 4369 |  |  |
|  | SK 014 | 9 | 0.67 | 2.67 | 0.00 | neg | 602 |  |  |  |
|  |  |  |  |  |  |  |  |  |  |  |
| **7** | SK 024 | Baseline | 9.67 | 7.33 | 3.00 | pos (ESAT6) | 225 |  |  |  |
|  | SK 024 | 3 | 28.67 | 13.00 | 0.00 | pos (ESAT6/CFP10) | 343 | 295 | Yes |  |
|  | SK 024 | 6 | 18.33 | 5.00 | 0.00 | pos (ESAT6/CFP10) | 340 |  | Yes |  |
|  | SK 024 | 9 | 149.67 | 10.00 | 0.33 | pos (ESAT6/CFP10) | 400 |  | Yes |  |
|  | SK 024 | 12 | 0.00 | 10.67 | 0.00 | pos (CFP10) | 339 |  | Yes |  |
|  | SK 024 | 15 | 12.00 | 2.67 | 0.33 | pos (ESAT6) | 356 | 146 | Yes |  |
|  |  |  |  |  |  |  |  |  |  |  |
| **8** | SK 026 | Baseline | 35.67 | 10.33 | 1.00 | pos (ESAT6/CFP10) | 570 | 7201 |  |  |
|  | SK 026 | 3 | 24.33 | 18.67 | 0.67 | pos (ESAT6/CFP10) | 622 |  |  |  |
|  | SK 026 | 6 | 4.00 | 8.67 | 0.00 | pos (CFP10) | 506 |  |  |  |
|  | SK 026 | 9 | 42.33 | 15.67 | 1.33 | pos (ESAT6/CFP10) | 656 |  |  |  |
|  | SK 026 | 12 | 9.67 | 0.33 | 0.67 | pos (ESAT6) | 399 | 43353 |  |  |
|  | SK 026 | 15 | 163.00 | 19.33 | 2.33 | pos (ESAT6/CFP10) | 429 |  |  |  |
|  | SK 026 | 21 | 13.67 | 1.67 | 0.00 | pos (ESAT6) | 445 |  |  |  |
|  |  |  |  |  |  |  |  |  |  |  |
| **9** | SK 034 | Baseline | 100.00 | 4.00 | 1.67 | pos (ESAT6) | 239 | 199534 |  |  |
|  | SK 034 | 3 | 13.00 | 3.67 | 0.67 | pos (ESAT6) | 242 |  |  |  |
|  | SK 034 | 6 | 29.33 | 3.67 | 0.00 | pos (ESAT6) | 323 | 209288 |  |  |
|  | SK 034 | 9 | BO | 92.33 | 75.67 | INDETERMINATE | 231 |  |  |  |
|  |  |  |  |  |  |  |  |  |  |  |
| **10** | SK 035 | Baseline | 0.67 | 0.00 | 0.00 | neg | 444 |  |  |  |
|  | SK 035 | 3 | 0.33 | 3.00 | 0.00 | neg | 469 | 24818 |  |  |
|  | SK 035 | 6 | 0.00 | 0.00 | 0.33 | neg | 544 |  |  |  |
|  | SK 035 | 9 | 0.33 | 0.00 | 0.00 | neg | 502 | 4101 |  |  |
|  |  |  |  |  |  |  |  |  |  |  |
| **11** | SK 036 | Baseline | 6.33 | 24.33 | 0.00 | pos (ESAT6/CFP10) | 511 |  |  |  |
|  | SK 036 | 3 | 0.33 | 4.67 | 0.67 | neg | 444 | 3744 |  |  |
|  | SK 036 | 6 | 1.33 | 83.33 | 0.00 | pos (CFP10) | 439 |  |  |  |
|  | SK 036 | 9 | 3.00 | 33.67 | 0.00 | pos (CFP10) | 363 | 9976 |  |  |
|  |  |  |  |  |  |  |  |  |  |  |
| **12** | SK 037 | Baseline | 0.67 | 1.33 | 0.33 | neg | 331 | 86636 |  |  |
|  | SK 037 | 3 | 0.00 | 1.00 | 0.67 | neg | 377 |  |  |  |
|  | SK 037 | 6 | 5.67 | 20.00 | 0.00 | pos (ESAT6/CFP10) | 518 | 61107 |  |  |
|  | SK 037 | 9 | 0.67 | 1.00 | 0.00 | neg | 283 |  |  |  |
|  |  |  |  |  |  |  |  |  |  |  |
| **13** | SK 047 | Baseline | 0.67 | 1.00 | 0.67 | neg | 439 |  |  |  |
|  | SK 047 | 3 | 1.00 | 0.00 | 0.33 | neg | 430 | 9546 |  |  |
|  | SK 047 | 6 | 4.33 | 1.00 | 0.00 | pos (ESAT6) | 471 |  |  |  |
|  | SK 047 | 9 | 4.67 | 0.33 | 0.00 | pos (ESAT6) | 570 | 6070 |  |  |
|  | SK 047 | 12 | 0.00 | 0.00 | 0.00 | INDETERMINATE | 452 |  |  |  |
|  |  |  |  |  |  |  |  |  |  |  |
| **14** | SK 050 | Baseline | 2.33 | 0.00 | 1.00 | neg | 433 |  |  |  |
|  | SK 050 | 3 | 0.33 | 0.00 | 0.00 | neg | 486 | 24148 |  |  |
|  | SK 050 | 6 | 0.00 | 0.67 | 0.00 | neg | 472 |  |  |  |
|  | SK 050 | 9 | 2.00 | 0.00 | 0.33 | neg | 510 | 24163 |  |  |
|  | SK 050 | 12 | 1.33 | 0.67 | 0.33 | neg | 489 | 26639 |  |  |
|  | SK 050 | 15 | 0.33 | 0.67 | 0.33 | neg | 484 | 16557 |  |  |
|  |  |  |  |  |  |  |  |  |  |  |
| **15** | SK 063 | Baseline | 0.33 | 0.67 | 0.33 | neg | 5 | 82483 |  |  |
|  | SK 063 | 3 | 0.00 | 0.00 | 0.00 | neg | 27 |  | Yes |  |
|  | SK 063 | 6 | 0.33 | 0.00 | 0.00 | neg | 46 |  | Yes |  |
|  | SK 063 | 9 | 0.00 | 0.67 | 0.33 | neg | 140 |  | Yes |  |
|  | SK 063 | 12 | 0.00 | 0.00 | 0.00 | INDETERMINATE | 154 | 0 | Yes |  |
|  |  |  |  |  |  |  |  |  |  |  |
| **16** | SK 066 | Baseline | 0.33 | 3.00 | 0.33 | neg | 151 | 18320 |  |  |
|  | SK 066 | 3 | 3.33 | 14.67 | 4.00 | pos (CFP10) | 177 |  | Yes |  |
|  | SK 066 | 6 | 0.33 | 34.00 | 0.00 | pos (CFP10) | 181 | 168484 | Yes |  |
|  | SK 066 | 9 | 4.67 | 86.00 | 0.00 | pos (ESAT6/CFP10) | 179 |  | Yes |  |
|  |  |  |  |  |  |  |  |  |  |  |
| **17** | SK 067 | Baseline | 63.00 | 18.00 | 1.67 | pos (ESAT6/CFP10) | 113 |  |  |  |
|  | SK 067 | 3 | 15.33 | 10.00 | 0.33 | pos (ESAT6/CFP10) | 135 | 93189 |  |  |
|  | SK 067 | 6 | 0.33 | 1.33 | 0.00 | neg | 125 |  |  |  |
|  |  |  |  |  |  |  |  |  |  |  |
| **18** | SK 068 | Baseline | 0.00 | 0.00 | 0.33 | neg | 579 |  |  |  |
|  | SK 068 | 3 | 5.67 | 23.67 | 0.00 | pos (ESAT6/CFP10) | 571 | 33695 |  |  |
|  | SK 068 | 6 | 2.67 | 13.67 | 0.00 | pos (CFP10) | 582 |  |  |  |
|  | SK 068 | 9 | 2.00 | 13.00 | 0.00 | pos (CFP10) | 534 |  |  |  |
|  | SK 068 | 12 | 3.33 | 136.33 | 0.00 | pos (CFP10) | 531 |  |  |  |
|  |  |  |  |  |  |  |  |  |  |  |
| **19** | SK 079 | Baseline | 31.00 | 16.33 | 2.67 | pos (ESAT6/CFP10) | 470 |  |  |  |
|  | SK 079 | 3 | 10.00 | 6.33 | 2.00 | pos (ESAT6/CFP10) | 462 |  |  |  |
|  | SK 079 | 6 | 0.00 | 0.33 | 0.00 | neg | 478 |  |  |  |
|  | SK 079 | 9 | 17.33 | 20.00 | 0.00 | pos (ESAT6/CFP10) | 501 | 79515 |  |  |
|  |  |  |  |  |  |  |  |  |  |  |
| **20** | SK 081 | Baseline | 2.00 | 0.67 | 2.67 | neg | 315 | 85288 |  |  |
|  | SK 081 | 3 | 0.33 | 0.67 | 0.00 | neg | 231 |  |  |  |
|  | SK 081 | 6 | 2.67 | 1.67 | 1.33 | neg | 206 | 302382 |  |  |
|  | SK 081 | 9 | 9.67 | 19.67 | 46.67 | INDETERMINATE | 103 | 252847 |  |  |
|  |  |  |  |  |  |  |  |  |  |  |
| **21** | SK 086 | Baseline | 6.00 | 36.33 | 1.67 | pos (ESAT6/CFP10) | 709 | 1483 |  |  |
|  | SK 086 | 3 | 1.00 | 24.67 | 0.33 | pos (CFP10) | 742 |  |  |  |
|  | SK 086 | 6 | 1.67 | 9.33 | 0.00 | pos (CFP10) | 827 |  |  |  |
|  | SK 086 | 9 | 0.33 | 30.67 | 0.00 | pos (CFP10) | 914 |  |  |  |
|  | SK 086 | 12 | 2.33 | 65.33 | 1.00 | pos (CFP10) | 916 | 1125 |  |  |
|  | SK 086 | 15 | 2.67 | 50.67 | 0.00 | pos (CFP10) | 732 |  |  |  |
|  | SK 086 | 21 | 1.00 | 47.00 | 0.00 | pos (CFP10) | 999 | 567 | **Yes- on PMTCT** |  |
|  |  |  |  |  |  |  |  |  |  |  |
| **22** | SK 088 | Baseline | 8.67 | 2.33 | 1.00 | pos (ESAT6) | 351 | 401 |  |  |
|  | SK 088 | 3 | 4.33 | 0.67 | 0.33 | neg | 274 |  |  |  |
|  | SK 088 | 6 | 19.67 | 10.33 | 1.00 | pos (ESAT6/CFP10) | 322 | 429 |  |  |
|  | SK 088 | 9 | 22.33 | 6.33 | 0.33 | pos (ESAT6/CFP10) | 315 |  |  |  |
|  | SK 088 | 12 | 28.67 | 9.33 | 0.00 | pos (ESAT6/CFP10) | 398 | 357 |  |  |
|  | SK 088 | 15 | 12.67 | 3.67 | 0.33 | pos (ESAT6) | 376 |  |  |  |
|  | SK 088 | 21 | 43.00 | 39.00 | 0.67 | pos (ESAT6/CFP10) | 292 | 689 |  |  |
|  |  |  |  |  |  |  |  |  |  |  |
| **23** | SK 098 | Baseline | 2.67 | 15.67 | 1.67 | pos (CFP10) | 371 | 57248 |  |  |
|  | SK 098 | 3 | 0.33 | 6.00 | 0.00 | pos (CFP10) | 293 |  |  |  |
|  | SK 098 | 6 | 0.33 | 3.33 | 0.00 | neg | 314 | 47901 |  |  |
|  | SK 098 | 9 | 2.67 | 9.33 | 0.67 | pos (CFP10) | 223 |  |  |  |
|  | SK 098 | 12 | 2.00 | 6.67 | 0.00 | pos (CFP10) | 211 | 93381 |  |  |
|  | SK 098 | 15 | 1.33 | 3.00 | 2.00 | neg | 287 |  | Yes |  |
|  | SK 098 | 21 | 0.33 | 9.67 | 0.00 | pos (CFP10) | 351 | 82 | Yes |  |
|  |  |  |  |  |  |  |  |  |  |  |
| **24** | SK 101 | Baseline | 22.67 | 4.00 | 1.00 | pos (ESAT6) | 119 |  |  |  |
|  | SK 101 | 3 | 3.67 | 1.00 | 1.00 | neg | 245 | 319813 |  |  |
|  | SK 101 | 6 | BO | 25.67 | 1.00 | pos (ESAT6/CFP10) | 146 |  |  |  |
|  |  | 9 |  |  |  |  |  |  |  |  |
| **25** | SK 103 | Baseline | 0.33 | 1.00 | 0.00 | neg | 1111 | 413600 |  |  |
|  | SK 103 | 3 | 5.67 | 16.67 | 1.33 | pos (ESAT6/CFP10) | 1127 |  |  |  |
|  | SK 103 | 6 | 1.67 | 6.33 | 1.67 | pos (CFP10) | 1617 | 25224 |  |  |
|  | SK 103 | 9 | 12.33 | 28.67 | 3.00 | pos (ESAT6/CFP10) | 1494 |  |  |  |
|  | SK 103 | 12 | 3.00 | 15.00 | 0.33 | pos (CFP10) | 1391 | 23326 |  |  |
|  |  |  |  |  |  |  |  |  |  |  |
| **26** | SK 111 | Baseline | 0.67 | 0.67 | 1.00 | neg | 458 | 299491 |  |  |
|  | SK 111 | 3 | 0.00 | 0.00 | 0.33 | neg | 272 |  |  |  |
|  | SK 111 | 6 | 0.33 | 1.33 | 0.00 | neg | 331 | 196533 |  |  |
|  | SK 111 | 9 | 1.00 | 0.00 | 0.33 | neg | 282 |  |  |  |
|  | SK 111 | 12 | 0.00 | 0.33 | 0.00 | neg | 617 | 365847 |  |  |
|  | SK 111 | 15 | 0.33 | 0.67 | 0.00 | neg | 218 |  |  |  |
|  | SK 111 | 21 | 0.00 | 0.67 | 0.33 | neg | 214 | 654608 |  |  |
|  |  |  |  |  |  |  |  |  |  |  |
| **27** | SK 113 | Baseline | 6.00 | 11.00 | 0.67 | pos (ESAT6/CFP10) | 426 |  |  |  |
|  | SK 113 | 3 | 6.33 | 20.33 | 0.00 | pos (ESAT6/CFP10) | 345 | < 40 |  |  |
|  | SK 113 | 6 | 3.33 | 9.67 | 0.00 | pos (CFP10) | 351 |  |  |  |
|  | SK 113 | 9 | 6.33 | 9.00 | 4.33 | neg | 314.58 | <40 |  |  |
|  | SK 113 | 12 | 1.67 | 31.67 | 0.33 | pos (CFP10) | 364 |  |  |  |
|  | SK 113 | 15 | 5.00 | 36.33 | 0.33 | pos (ESAT6/CFP10) |  |  |  |  |
|  |  |  |  |  |  |  |  |  |  |  |
| **28** | SK 114 | Baseline | 7.67 | 5.33 | 3.00 | neg | 252 |  |  |  |
|  | SK 114 | 3 | 0.33 | 0.00 | 0.33 | neg | 274 | 5070 |  |  |
|  | SK 114 | 6 | 0.67 | 0.33 | 0.33 | neg | 267 |  |  |  |
|  | SK 114 | 9 | 0.00 | 0.33 | 0.00 | neg | 337 | 17138 |  |  |
|  | SK 114 | 12 | 1.00 | 1.00 | 0.00 | neg | 317 |  |  |  |
|  |  |  |  |  |  |  |  |  |  |  |
| **29** | SK 121 | Baseline | 3.67 | 10.00 | 4.67 | neg | 110 | 735592 |  |  |
|  | SK 121 | 3 | 1.33 | 3.33 | 0.33 | neg | 95 |  |  |  |
|  | SK 121 | 6 | 1.00 | 1.33 | 1.00 | INDETERMINATE | 136 |  |  |  |
|  | SK 121 | 9 | 6.33 | 9.00 | 4.33 | neg | 187 |  | Yes |  |
|  | SK 121 | 12 | 0.00 | 0.00 | 0.00 | INDETERMINATE | 138 | 253936 | Yes |  |
|  |  |  |  |  |  |  |  |  |  |  |
| **30** | SK 123 | Baseline | 5.67 | 4.67 | 0.00 | pos (ESAT6/CFP10) | 231 |  |  |  |
|  | SK 123 | 3 | 1.00 | 1.67 | 0.00 | neg | 167 |  |  |  |
|  | SK 123 | 6 | 0.00 | 0.00 | 0.00 | neg | 259 |  |  |  |
|  |  |  |  |  |  |  |  |  |  |  |
| **31** | SK 135 | Baseline | 24.00 | 11.67 | 3.33 | pos (ESAT6/CFP10) | 233 | 20158 |  |  |
|  | SK 135 | 3 | 9.00 | 6.00 | 2.00 | pos (ESAT6) | 245 |  |  |  |
|  | SK 135 | 6 | 0.00 | 0.00 | 0.00 | INDETERMINATE | 210.68 | 12727 |  |  |
|  |  |  |  |  |  |  |  |  |  |  |
| **32** | SK 138 | Baseline | 3.00 | 4.00 | 0.33 | neg | 233 |  |  |  |
|  | SK 138 | 3 | 1.67 | 1.33 | 0.33 | neg | 238 | 13213 |  |  |
|  | SK 138 | 6 | 2.00 | 1.00 | 0.33 | neg | 231 |  |  |  |
|  | SK 138 | 9 | 0.33 | 2.33 | 0.00 | neg | 284 | 19699 |  |  |
|  |  |  |  |  |  |  |  |  |  |  |
| **33** | SK 139 | Baseline | 2.67 | 1.33 | 0.00 | neg | 183 | 47547 |  |  |
|  | SK 139 | 3 | 1.67 | 1.67 | 0.00 | neg | 184 |  |  |  |
|  | SK 139 | 6 | 15.33 | 2.00 | 0.00 | pos (ESAT6) | 161 | 23165 |  |  |
|  | SK 139 | 9 | 26.67 | 4.00 | 0.67 | pos (ESAT6) | 128 |  |  |  |
|  |  |  |  |  |  |  |  |  |  |  |
| **34** | SK 141 | Baseline | 14.33 | 15.00 | 1.00 | pos (ESAT6/CFP10) | 392 |  |  |  |
|  | SK 141 | 3 | 4.00 | 12.00 | 0.33 | pos (CFP10) | 418 | 2676 |  |  |
|  | SK 141 | 6 | 5.67 | 18.00 | 0.33 | pos (ESAT6/CFP10) | 278 |  |  |  |
|  | SK 141 | 9 | 5.00 | BO | 2.00 | pos (CFP10) | 277 |  |  |  |
|  | SK 141 | 12 | 0.67 | 7.67 | 0.00 | pos (CFP10) | 293 |  |  |  |
|  | SK 141 | 15 | 1.33 | 4.67 | 0.00 | pos (CFP10) | 325 | 1806 |  |  |
|  |  |  |  |  |  |  |  |  |  |  |
| **35** | SK 142 | Baseline | 5.00 | 1.33 | 0.67 | pos (ESAT6) | 275 |  |  |  |
|  | SK 142 | 3 | 11.00 | 3.33 | 0.33 | pos (ESAT6) | 238 |  |  |  |
|  | SK 142 | 6 | 5.00 | 4.33 | 0.00 | pos (ESAT6/CFP10) | 384 |  | Yes |  |
|  | SK 142 | 9 | 3.00 | 0.67 | 0.00 | neg | 350 |  | Yes |  |
|  | SK 142 | 12 | 7.67 | 0.67 | 0.00 | pos (ESAT6) | 348 | <40 | Yes |  |
|  |  |  |  |  |  |  |  |  |  |  |
| **36** | SK 150 | Baseline | 10.67 | 13.33 | 4.67 | neg | 255 |  |  |  |
|  | SK 150 | 3 | 17.33 | 18.00 | 0.00 | pos (ESAT6/CFP10) | 222 |  |  |  |
|  | SK 150 | 6 | 14.00 | 17.33 | 0.00 | pos (ESAT6/CFP10) | 216 |  |  |  |
|  | SK 150 | 9 | 88.00 | 20.00 | 0.00 | pos (ESAT6/CFP10) | 281 |  |  |  |
|  | SK 150 | 12 | 47.67 | 6.67 | 0.00 | pos (ESAT6/CFP10) | 250 | 148065 |  |  |
|  |  |  |  |  |  |  |  |  |  |  |
| **37** | SK 155 | Baseline | 7.00 | 0.33 | 0.33 | pos (ESAT6) | 518 | 9239 |  |  |
|  | SK 155 | 3 | 7.00 | 1.67 | 1.67 | pos (ESAT6) | 519 |  |  |  |
|  | SK 155 | 6 | 4.00 | 13.00 | 1.00 | pos (CFP10) | 472 | 19652 |  |  |
|  | SK 155 | 9 | 2.67 | 0.33 | 0.33 | neg | 439 |  |  |  |
|  | SK 155 | 12 | 8.33 | 1.00 | 0.00 | pos (ESAT6) | 428 | 26973 |  |  |
|  | SK 155 | 15 | 1.67 | 0.67 | 0.33 | neg | 416 |  |  |  |
|  | SK 155 | 21 | 2.33 | 0.00 | 0.00 | neg | 397 | 83723 |  |  |
|  | SK 155 | 24 | 21.00 | 3.33 | 0.00 | pos (ESAT6) | 421 |  |  |  |
|  |  |  |  |  |  |  |  |  |  |  |
| **38** | SK 157 | Baseline | 0.00 | 41.67 | 26.00 | INDETERMINATE | 420 |  |  |  |
|  | SK 157 | 3 | 0.67 | 5.33 | 0.00 | pos (CFP10) | 397 | < 40.0 |  |  |
|  | SK 157 | 6 | 2.00 | 10.00 | 1.00 | pos (CFP10) | 467 |  |  |  |
|  | SK 157 | 9 | 5.67 | 13.33 | 0.67 | pos (ESAT6/CFP10) | 347 |  |  |  |
|  | SK 157 | 12 | 3.33 | 5.67 | 0.00 | pos (CFP10) | 335 |  |  |  |
|  |  |  |  |  |  |  |  |  |  |  |
| **39** | SK 163 | Baseline | 9.33 | 6.00 | 2.33 | pos (ESAT6) | 622 | 21895 |  |  |
|  | SK 163 | 3 | 7.67 | 2.33 | 0.67 | pos (ESAT6) | 520 |  |  |  |
|  | SK 163 | 6 | 19.67 | 3.67 | 0.33 | pos (ESAT6) | 613 | 16542 |  |  |
|  | SK 163 | 9 | 6.33 | 2.00 | 0.00 | pos (ESAT6) | 526 |  |  |  |
|  |  |  |  |  |  |  |  |  |  |  |
| **40** | SK 167 | Baseline | 0.00 | 1.67 | 1.33 | neg | 397 |  |  |  |
|  | SK 167 | 3 | 0.33 | 0.00 | 0.00 | neg | 542 | 19884 |  |  |
|  | SK 167 | 6 | 0.33 | 0.33 | 0.00 | neg | 566 |  |  |  |
|  | SK 167 | 9 | 7.33 | 5.33 | 9.00 | neg | 448 | 24309 |  |  |
|  | SK 167 | 12 | 28.00 | 45.33 | 34.00 | INDETERMINATE | 443 |  |  |  |
|  |  |  |  |  |  |  |  |  |  |  |
| **41** | SK 169 | Baseline | 13.00 | 17.67 | 9.33 | neg | 371 | 1536 |  |  |
|  | SK 169 | 3 | 3.00 | 1.67 | 1.00 | neg | 439 |  |  |  |
|  | SK 169 | 6 | 2.00 | 5.00 | 1.33 | neg | 325 | 4186 |  |  |
|  | SK 169 | 9 | 8.33 | 8.00 | 3.00 | neg | 327 |  |  | Yes (treatment initiated 4 weeks previously) |
|  | SK 169 | 12 | 1.00 | 2.33 | 0.33 | neg | 586 | 4052 |  | Yes |
|  |  |  |  |  |  |  |  |  |  |  |
| **42** | SK 171 | Baseline | 16.67 | 2.33 | 0.33 | pos (ESAT6) | 101 | 349937 |  |  |
|  | SK 171 | 3 | 23.00 | 16.67 | 19.67 | INDETERMINATE | 34 |  |  |  |
|  |  |  |  |  |  |  |  |  |  |  |
| **43** | SK 179 | Baseline | 35.00 | 38.00 | 38.33 | INDETERMINATE | 339 |  |  |  |
|  | SK 179 | 3 | BO | BO | BO | INDETERMINATE | 302 |  |  |  |
|  | SK 179 | 6 | BO | BO | BO | INDETERMINATE | 349 | 118131 |  |  |
|  |  |  |  |  |  |  |  |  |  |  |
| **44** | SK 180 | Baseline | 0.00 | 0.33 | 0.00 | neg | 414 |  |  |  |
|  | SK 180 | 3 | 1.33 | 2.67 | 0.33 | neg | 392 | 6704 |  |  |
|  | SK 180 | 6 | 0.67 | 0.33 | 0.00 | neg | 248 |  |  |  |
|  |  |  |  |  |  |  |  |  |  |  |
| **45** | SK 186 | Baseline | 6.67 | 7.00 | 2.00 | pos (ESAT6/CFP10) | 254 |  |  |  |
|  | SK 186 | 3 | 22.33 | 16.67 | 21.33 | INDETERMINATE | 153 | 29812 |  |  |
|  | SK 186 | 6 | 2.67 | 3.67 | 1.00 | neg | 230 |  |  |  |
|  | SK 186 | 9 | 1.00 | 0.67 | 0.00 | neg | 207 |  |  |  |
|  |  |  |  |  |  |  |  |  |  |  |
| **46** | SK 187 | Baseline | 1.67 | 4.33 | 1.67 | neg | 627 |  |  |  |
|  | SK 187 | 3 | 18.67 | 33.00 | 14.33 | INDETERMINATE | 548 | 7499 |  |  |
|  |  |  |  |  |  |  |  |  |  |  |
| **47** | SK 188 | Baseline | 20.67 | 25.00 | 0.00 | pos (ESAT6/CFP10) | 717 | 8279 |  |  |
|  | SK 188 | 3 | 0.00 | 0.00 | 0.00 | INDETERMINATE | 519 |  |  |  |
|  | SK 188 | 6 | BO | BO | BO | INDETERMINATE | 692 | <40 | Yes |  |
|  | SK 188 | 9 | BO | BO | BO | INDETERMINATE | 783 |  | Yes |  |
|  |  |  |  |  |  |  |  |  |  |  |
| **48** | SK 195 | Baseline | 17.33 | 6.00 | 2.67 | pos (ESAT6) | 171 | 30553 |  |  |
|  | SK 195 | 3 | 9.00 | 1.00 | 0.33 | pos (ESAT6) | 207 |  |  |  |
|  | SK 195 | 6 | 13.33 | 0.00 | 0.00 | pos (ESAT6) | 255 | 10077 |  |  |
|  | SK 195 | 9 | 11.33 | 0.67 | 0.00 | pos (ESAT6) | 209 |  |  |  |
|  |  |  |  |  |  |  |  |  |  |  |
| **49** | SK 199 | Baseline | 13.00 | 23.67 | 11.33 | INDETERMINATE | 515 |  |  |  |
|  | SK 199 | 3 | 11.00 | 16.00 | 9.67 | neg | 598 | 265 |  |  |
|  | SK 199 | 6 | 12.67 | 29.00 | 3.00 | pos (ESAT6/CFP10) | 437 |  |  |  |
|  | SK 199 | 9 | 1.33 | 10.00 | 0.00 | pos (CFP10) | 552 | <40 | **Yes- on PMTCT** |  |
|  | SK 199 | 12 | 6.67 | 13.67 | 2.33 | pos (CFP10) | 660 |  |  |  |
|  |  |  |  |  |  |  |  |  |  |  |
| **50** | SK 200 | Baseline | 0.00 | 0.00 | 0.00 | INDETERMINATE | 557 |  |  |  |
|  | SK 200 | 3 | 0.00 | 0.00 | 0.00 | neg | 622 |  |  |  |
|  | SK 200 | 6 | 0.00 | 0.00 | 0.00 | INDETERMINATE | 433 | 16476 |  |  |
|  |  |  |  |  |  |  |  |  |  |  |
| **51** | SK 201 | Baseline | 3.00 | 3.00 | 1.33 | neg | 310 | 216478 |  |  |
|  | SK 201 | 3 | 8.33 | 4.00 | 1.67 | pos (ESAT6) | 292 |  |  |  |
|  | SK 201 | 6 | 13.00 | 4.00 | 1.67 | pos (ESAT6) | 299 | 279831 |  |  |
|  | SK 201 | 9 | 21.00 | 16.67 | 1.33 | pos (ESAT6/CFP10) | 319 |  |  |  |
|  | SK 201 | 12 | 27.33 | 19.67 | 10.67 | INDETERMINATE | 207 | 29875 |  |  |
|  |  |  |  |  |  |  |  |  |  |  |
| **52** | SK 202 | Baseline | 4.00 | 9.00 | 0.33 | pos (CFP10) | 223 |  |  |  |
|  | SK 202 | 3 | 2.33 | 6.67 | 0.00 | pos (CFP10) | 176 | 391422 |  |  |
|  | SK 202 | 6 | 0.33 | 1.33 | 0.33 | neg | 250 |  | Yes |  |
|  | SK 202 | 9 | BO | BO | BO | INDETERMINATE | 397 | 74 | Yes |  |
|  |  |  |  |  |  |  |  |  |  |  |
| **53** | SK 208 | Baseline | 1.67 | 2.00 | 1.00 | neg | 101 |  |  |  |
|  | SK 208 | 3 | 3.67 | 0.33 | 0.00 | neg | 126 | 25523 |  |  |
|  | SK 208 | 6 | 0.33 | 0.00 | 0.33 | neg | 122 |  |  |  |
|  | SK 208 | 9 | 0.00 | 0.33 | 0.00 | neg | 52 | 45054 |  |  |
|  | SK 208 | 12 | 5.00 | 8.33 | 10.00 | neg | 58 |  | Yes |  |
|  |  |  |  |  |  |  |  |  |  |  |
| **54** | SK 209 | Baseline | 35.00 | 36.00 | 56.33 | INDETERMINATE | 366 | 722 |  |  |
|  | SK 209 | 3 | 3.33 | 7.67 | 6.67 | neg | 370 |  |  |  |
|  | SK 209 | 6 | 0.67 | 2.00 | 1.67 | neg | 325 | 1140 |  |  |
|  | SK 209 | 9 | 5.00 | 7.00 | 0.67 | pos (ESAT6/CFP10) | 516 |  |  |  |
|  | SK 209 | 12 | 13.33 | 13.67 | 11.67 | INDETERMINATE | 426 | 448 |  |  |
|  |  |  |  |  |  |  |  |  |  |  |
| **55** | SK 212 | Baseline | 7.67 | 4.33 | 0.00 | pos (ESAT6/CFP10) | 347 | 19670 |  |  |
|  | SK 212 | 3 | 1.33 | 0.33 | 0.00 | neg | 228 |  |  |  |
|  | SK 212 | 6 | 5.67 | 2.00 | 3.67 | neg | 258 | 59344 |  |  |
|  | SK 212 | 9 | 3.00 | 1.67 | 0.33 | neg | 279 |  |  |  |
|  |  |  |  |  |  |  |  |  |  |  |
| **56** | SK 213 | Baseline | 2.33 | 0.67 | 2.67 | neg | 182 |  |  |  |
|  | SK 213 | 3 | 0.67 | 1.67 | 0.33 | neg | 139 | 4286 |  |  |
|  |  |  |  |  |  |  |  |  |  |  |
| **57** | SK 214 | Baseline | 29.00 | 50.33 | 2.67 | pos (ESAT6/CFP10) | 169 | 11492 |  |  |
|  | SK 214 | 3 | 27.00 | 26.00 | 2.67 | pos (ESAT6/CFP10) | 182 |  | Yes |  |
|  | SK 214 | 6 | 2.00 | 55.00 | 1.67 | pos (CFP10) | 236 | < 40.0 | Yes |  |
|  | SK 214 | 9 | 0.00 | 0.00 | 0.00 | INDETERMINATE | 254 |  | Yes |  |
|  |  |  |  |  |  |  |  |  |  |  |
| **58** | SK 218 | Baseline | 14.67 | 4.33 | 1.67 | pos (ESAT6) | 167 | 29681 |  |  |
|  | SK 218 | 3 | 1.67 | 1.00 | 0.33 | neg | 264 |  |  |  |
|  | SK 218 | 6 | 4.00 | 1.00 | 0.33 | neg | 213 |  | Yes |  |
|  | SK 218 | 9 | 2.67 | 0.00 | 0.00 | neg | 425 | 695 | Yes |  |
|  |  |  |  |  |  |  |  |  |  |  |
| **59** | SK 221 | Baseline | 1.00 | 2.00 | 0.00 | neg | 333 |  |  |  |
|  | SK 221 | 3 | 0.67 | 0.67 | 0.00 | neg | 298 |  |  |  |
|  | SK 221 | 6 | 0.00 | 0.00 | 0.00 | neg | 298 |  |  |  |
|  | SK 221 | 9 | 0.33 | 0.00 | 0.00 | neg | 302 | 27088 |  |  |
|  |  |  |  |  |  |  |  |  |  |  |
| **60** | SK 224 | Baseline | 16.33 | 26.33 | 1.67 | pos (ESAT6/CFP10) | 267 |  |  |  |
|  | SK 224 | 3 | 0.67 | 5.00 | 0.67 | neg | 284 | 468498 |  |  |
|  | SK 224 | 6 | 1.00 | 2.00 | 0.33 | neg | 209 |  |  |  |
|  | SK 224 | 9 | 0.00 | 1.67 | 0.00 | neg | 273 | 134819 |  |  |
|  | SK 224 | 12 | 1.33 | 3.33 | 1.00 | neg | 225 |  |  |  |
|  | SK 224 | 15 | 7.00 | 14.67 | 0.67 | pos (ESAT6/CFP10) | 192 | 289016 |  |  |
|  |  |  |  |  |  |  |  |  |  |  |
| **61** | SK 226 | Baseline | 2.00 | 4.00 | 0.00 | neg | 402 | 25032 |  |  |
|  | SK 226 | 3 | 22.67 | 4.00 | 0.67 | pos (ESAT6) | 397 |  |  |  |
|  | SK 226 | 6 | 22.67 | 7.33 | 1.33 | pos (ESAT6/CFP10) | 408 | 10651 |  |  |
|  |  |  |  |  |  |  |  |  |  |  |
| **62** | SK 232 | Baseline | 8.33 | 18.00 | 1.00 | pos (ESAT6/CFP10) | 228 |  |  |  |
|  | SK 232 | 3 | 9.33 | 22.67 | 0.00 | pos (ESAT6/CFP10) | 260 | 21619 |  |  |
|  | SK 232 | 6 | 1.00 | 8.33 | 0.00 | neg | 274 |  |  |  |
|  | SK 232 | 9 | 0.00 | 0.00 | 0.00 | INDETERMINATE | 248 | 23538 |  |  |
|  | SK 232 | 12 | 0.00 | 0.00 | 0.00 | INDETERMINATE | 197 |  |  |  |
|  | SK 232 | 15 | 1.00 | 8.00 | 1.67 | pos (CFP10) | 316 |  |  |  |
|  |  |  |  |  |  |  |  |  |  |  |
| **63** | SK 235 | Baseline | 9.00 | 11.67 | 1.00 | pos (ESAT6/CFP10) | 475 | 554 |  |  |
|  | SK 235 | 3 | 15.67 | 12.67 | 0.00 | pos (ESAT6/CFP10) | 377 |  |  |  |
|  | SK 235 | 6 | 78.67 | 23.67 | 2.00 | pos (ESAT6/CFP10) | 488 |  |  |  |
|  | SK 235 | 9 | 27.00 | 22.33 | 0.00 | pos (ESAT6/CFP10) | 485 | 554 |  |  |
|  |  |  |  |  |  |  |  |  |  |  |
| **64** | SK 236 | Baseline | 4.67 | 26.33 | 0.00 | pos (ESAT6/CFP10) | 268 |  |  |  |
|  | SK 236 | 3 | 2.67 | 18.33 | 8.00 | pos (ESAT6/CFP10) | 184 | 272227 |  |  |
|  | SK 236 | 6 | 1.00 | 14.67 | 0.00 | pos (CFP10) | 117 |  |  |  |
|  | SK 236 | 9 | BO | BO | BO | INDETERMINATE | 73 | 182769 |  |  |
|  | SK 236 | 12 | 0.33 | 81.67 | 0.00 | pos (CFP10) | 56 |  |  | Yes (treatment initiated 8 weeks previously) |
|  |  |  |  |  |  |  |  |  |  |  |
| **65** | SK 242 | Baseline | 4.67 | 4.00 | 2.00 | neg | 523 | 723 |  |  |
|  | SK 242 | 3 | 7.00 | 5.33 | 3.00 | neg | 431 |  |  |  |
|  | SK 242 | 6 | 1.00 | 1.33 | 0.00 | neg | 654 | 763 |  |  |
|  | SK 242 | 9 | 10.00 | 7.33 | 0.00 | pos (ESAT6/CFP10) | 556 |  |  |  |
|  | SK 242 | 12 | 8.33 | 3.00 | 1.00 | pos (ESAT6) | 584 | 76 |  |  |
|  |  |  |  |  |  |  |  |  |  |  |
| **66** | SK 244 | Baseline | 7.00 | 7.00 | 5.33 | neg | 192 |  |  |  |
|  | SK 244 | 3 | 1.00 | 0.33 | 0.33 | neg | 256 | 4072 |  |  |
|  | SK 244 | 6 | 1.00 | 0.33 | 0.67 | neg | 294 |  |  |  |
|  | SK 244 | 9 | 1.00 | 1.00 | 0.00 | neg | 236 | 16114 |  |  |
|  | SK 244 | 12 | 14.00 | 13.33 | 24.00 | INDETERMINATE | 239 |  |  |  |
|  |  |  |  |  |  |  |  |  |  |  |
| **67** | SK 246 | Baseline | 1.00 | 0.00 | 0.67 | neg | 204 |  |  |  |
|  | SK 246 | 3 | 3.67 | 3.33 | 0.33 | neg | 476 | 128 | Yes |  |
|  | SK 246 | 6 | 2.00 | 2.33 | 0.00 | neg | 462 |  | Yes |  |
|  | SK 246 | 9 | 2.33 | 0.33 | 0.00 | neg | 664 | 104 | Yes |  |
|  | SK 246 | 12 | 1.00 | 0.33 | 0.33 | neg | 549 |  | Yes |  |
|  | SK 246 | 15 | 14.67 | 1.33 | 0.67 | pos (ESAT6) | 664 | 41 | Yes |  |
|  | SK 246 | 21 | 3.33 | 6.33 | 0.00 | pos (CFP10) | 602 |  | Yes |  |
|  |  |  |  |  |  |  |  |  |  |  |
| **68** | SK 247 | Baseline | 1.67 | 1.00 | 0.00 | neg | 326 |  |  |  |
|  | SK 247 | 3 | 0.33 | 0.33 | 0.33 | neg | 331 | 77989 |  |  |
|  | SK 247 | 6 | 3.33 | 0.33 | 0.33 | neg | 365 |  |  |  |
|  |  |  |  |  |  |  |  |  |  |  |
| **69** | SK 248 | Baseline | 1.00 | 8.67 | 0.00 | pos (CFP10) | 368 |  |  |  |
|  | SK 248 | 3 | 7.00 | 52.67 | 0.67 | pos (ESAT6/CFP10) | 401 | 407 |  |  |
|  | SK 248 | 6 | 3.00 | 7.00 | 0.67 | pos (CFP10) | 396 |  |  |  |
|  | SK 248 | 9 | 1.33 | 20.00 | 1.33 | pos (CFP10) | 416 | 850 |  |  |
|  |  |  |  |  |  |  |  |  |  |  |
| **70** | SK 257 | Baseline | 1.33 | 0.67 | 0.00 | neg | 330 | 21673 |  |  |
|  | SK 257 | 3 | 1.00 | 0.33 | 0.00 | neg | 329 |  |  |  |
|  | SK 257 | 6 | 0.00 | 0.00 | 0.00 | neg | 234 | 17087 |  |  |
|  | SK 257 | 9 | 0.00 | 0.00 | 0.00 | neg | 217 |  |  |  |
|  |  |  |  |  |  |  |  |  |  |  |
| **71** | SK 259 | Baseline | 14.00 | 14.00 | 4.00 | pos (ESAT6/CFP10) | 269 |  |  |  |
|  | SK 259 | 3 | 3.67 | 16.67 | 0.67 | pos (CFP10) | 267 | 139519 |  |  |
|  | SK 259 | 6 | 3.00 | 5.33 | 1.67 | neg | 307 |  |  |  |
|  | SK 259 | 9 | 5.00 | 3.67 | 8.00 | neg | 261 | 44310 |  |  |
|  | SK 259 | 12 | 0.00 | 0.00 | 0.00 | INDETERMINATE | 247 |  |  |  |
|  |  |  |  |  |  |  |  |  |  |  |
| **72** | SK 261 | Baseline | 7.00 | 1.67 | 2.00 | pos (ESAT6) | 361 |  |  |  |
|  | SK 261 | 3 | 7.33 | 3.00 | 1.33 | pos (ESAT6) | 494 |  |  |  |
|  | SK 261 | 6 | 4.67 | 9.33 | 0.00 | pos (ESAT6/CFP10) | 507 | 6123 |  |  |
|  | SK 261 | 9 | 1.67 | 1.00 | 0.00 | neg | 678 |  |  |  |
|  |  |  |  |  |  |  |  |  |  |  |
| **73** | SK 263 | Baseline | 31.67 | 4.33 | 5.33 | pos (ESAT6) | 301 | 46097 |  |  |
|  | SK 263 | 3 | 0.33 | 1.67 | 1.00 | neg | 306 |  |  |  |
|  | SK 263 | 6 | 1.00 | 1.67 | 1.00 | neg | 329 | 35155 |  |  |
|  | SK 263 | 9 | 0.00 | 0.00 | 0.00 | neg | 335 |  |  |  |
|  | SK 263 | 12 | 0.00 | 0.00 | 0.00 | INDETERMINATE | 232 | 48041 |  |  |
|  |  |  |  |  |  |  |  |  |  |  |
| **74** | SK 265 | Baseline | 5.67 | 1.67 | 0.00 | pos (ESAT6) | 693 | 3901 |  |  |
|  | SK 265 | 3 | 1.67 | 1.67 | 0.00 | neg | 844 |  |  |  |
|  | SK 265 | 6 | 5.33 | 4.33 | 0.00 | pos (ESAT6/CFP10) | 835 |  |  |  |
|  | SK 265 | 9 | 3.67 | 0.00 | 0.33 | neg | 836 |  |  |  |
|  |  |  |  |  |  |  |  |  |  |  |
| **75** | SK 272 | Baseline | 1.33 | 1.67 | 0.33 | neg | 1142 | 23414 |  |  |
|  | SK 272 | 3 | 2.33 | 0.00 | 0.00 | neg | 368 |  |  |  |
|  | SK 272 | 6 | 1.33 | 1.00 | 0.67 | neg | 437 | 11217 |  |  |
|  | SK 272 | 9 | 0.67 | 0.67 | 0.00 | neg | 465 |  |  |  |
|  | SK 272 | 12 | 0.00 | 0.00 | 0.33 | neg | 349 | 19313 |  |  |
|  | SK 272 | 15 | 0.33 | 1.33 | 0.00 | neg | 408 | 21116 |  |  |
|  |  |  |  |  |  |  |  |  |  |  |
| **76** | SK 275 | Baseline | 1.67 | 0.67 | 1.00 | neg | 507 | < 40.0 |  |  |
|  | SK 275 | 3 | 0.00 | 0.33 | 0.00 | neg | 542 |  |  |  |
|  | SK 275 | 6 | 0.00 | 0.00 | 0.00 | neg | 566 | < 40.0 |  |  |
|  |  |  |  |  |  |  |  |  |  |  |
| **77** | SK 276 | Baseline | 0.00 | 1.00 | 0.00 | neg | 365 | 148527 |  |  |
|  | SK 276 | 3 | 1.67 | 0.33 | 0.33 | neg | 556 |  |  |  |
|  | SK 276 | 6 | 0.33 | 0.00 | 0.00 | neg | 708 | 277262 |  |  |
|  | SK 276 | 9 | 0.00 | 0.00 | 0.67 | neg | 442 |  |  |  |
|  | SK 276 | 12 | 0.00 | 0.00 | 0.33 | neg | 462 | 251556 |  |  |
|  | SK 276 | 15 | 0.00 | 0.00 | 0.00 | neg | 499 |  |  |  |
|  | SK 276 | 21 | 0.00 | 0.00 | 0.00 | neg | 583 | 187651 |  |  |
|  |  |  |  |  |  |  |  |  |  |  |
| **78** | SK 278 | Baseline | 1.67 | 2.33 | 0.33 | neg | 300 |  |  |  |
|  | SK 278 | 3 | 0.33 | 3.33 | 0.33 | neg | 443 | 26036 |  |  |
|  | SK 278 | 6 | 2.33 | 2.00 | 1.00 | neg | 318 |  |  |  |
|  | SK 278 | 9 | 3.67 | 3.00 | 0.67 | neg | 350 | 10379 |  |  |
|  | SK 278 | 12 | 0.00 | 0.00 | 0.00 | neg | 388 |  |  |  |
|  | SK 278 | 15 | 0.00 | 0.00 | 0.00 | INDETERMINATE | 350 |  |  |  |
|  |  |  |  |  |  |  |  |  |  |  |
| **79** | SK 282 | Baseline | 26.67 | 16.00 | 2.00 | pos (ESAT6/CFP10) | 521 |  |  |  |
|  | SK 282 | 3 | 7.00 | 9.67 | 0.00 | pos (ESAT6/CFP10) | 490 | 118 |  |  |
|  | SK 282 | 6 | 6.33 | 12.33 | 0.00 | pos (ESAT6/CFP10) | 585 | 58 |  |  |
|  | SK 282 | 9 | 20.00 | 12.33 | 0.67 | pos (ESAT6/CFP10) | 569 |  |  |  |
|  | SK 282 | 12 | 31.33 | 34.67 | 2.33 | pos (ESAT6/CFP10) | 684 |  |  |  |
|  | SK 282 | 15 | 160.33 | 40.00 | 0.00 | pos (ESAT6/CFP10) | 584 | 0 |  |  |
|  |  |  |  |  |  |  |  |  |  |  |
| **80** | SK 283 | Baseline | 17.67 | 12.33 | 6.33 | neg | 310 |  |  |  |
|  | SK 283 | 3 | 3.67 | 3.67 | 1.33 | neg |  | 30504 |  |  |
|  | SK 283 | 6 | 7.33 | 3.00 | 0.00 | pos (ESAT6) | 330 |  |  |  |
|  | SK 283 | 9 | 6.67 | 7.00 | 2.00 | pos (ESAT6/CFP10) | 239 |  |  |  |
|  | SK 283 | 12 | 7.00 | 5.00 | 2.67 | neg | 292 |  |  |  |
|  | SK 283 | 15 | 3.67 | 2.33 | 0.00 | neg | 241 | 119117 |  |  |
|  |  |  |  |  |  |  |  |  |  |  |
| **81** | SK 287 | Baseline | 9.00 | 7.33 | 3.00 | neg | 502 |  |  |  |
|  | SK 287 | 3 | 3.33 | 1.00 | 0.33 | neg | 406 | 87251 |  |  |
|  | SK 287 | 6 | 3.33 | 0.67 | 0.33 | neg | 389 |  |  |  |
|  | SK 287 | 9 | 0.33 | 0.33 | 0.00 | neg | 290 | 31648 |  |  |
|  | SK 287 | 12 | 1.67 | 0.67 | 0.00 | neg | 316 |  |  |  |
|  |  |  |  |  |  |  |  |  |  |  |
| **82** | SK 291 | Baseline | 1.00 | 0.33 | 0.33 | neg | 316 |  |  |  |
|  | SK 291 | 3 | 2.67 | 1.67 | 0.00 | neg | 262 | 3133 |  |  |
|  | SK 291 | 6 | 4.33 | 2.33 | 0.00 | pos (ESAT6) | 249 |  |  |  |
|  | SK 291 | 9 | 1.00 | 2.67 | 0.00 | neg | 288 | 3229 |  |  |
|  |  |  |  |  |  |  |  |  |  |  |
| **83** | SK 292 | Baseline | 1.33 | 0.67 | 0.00 | neg | 570 |  |  |  |
|  | SK 292 | 3 | 0.33 | 0.67 | 0.00 | neg | 591 | 2954 |  |  |
|  | SK 292 | 6 | 0.33 | 0.67 | 0.00 | neg | 597 |  |  |  |
|  |  |  |  |  |  |  |  |  |  |  |
| **84** | SK 302 | Baseline | 32.67 | 12.33 | 0.67 | pos (ESAT6/CFP10) | 316 | 1772 |  |  |
|  | SK 302 | 3 | 0.33 | 2.67 | 5.33 | neg | 383 |  |  |  |
|  | SK 302 | 6 | 28.00 | 2.33 | 0.67 | pos (ESAT6) | 344 | 10553 |  |  |
|  | SK 302 | 9 | 32.00 | 11.67 | 3.00 | pos (ESAT6/CFP10) | 347 |  |  |  |
|  | SK 302 | 12 | 76.00 | 20.33 | 1.00 | pos (ESAT6/CFP10) | 324 | 12415 |  |  |
|  |  |  |  |  |  |  |  |  |  |  |
| **85** | SK 306 | Baseline | 5.00 | 56.00 | 1.00 | pos (CFP10) | 461 |  |  |  |
|  | SK 306 | 3 | 7.33 | 18.00 | 0.33 | pos (ESAT6/CFP10) | 622 | 20690 |  |  |
|  | SK 306 | 6 | 0.33 | 6.67 | 0.00 | pos (CFP10) | 402 |  |  |  |
|  | SK 306 | 9 | 0.33 | 7.33 | 0.00 | pos (CFP10) | 614 | 14058 |  |  |
|  | SK 306 | 12 | 17.67 | 29.33 | 17.33 | INDETERMINATE | 486 |  |  |  |
|  |  |  |  |  |  |  |  |  |  |  |
| **86** | SK 307 | Baseline | 1.33 | 1.67 | 0.33 | neg | 530 | 104829 |  |  |
|  | SK 307 | 3 | 3.00 | 2.00 | 0.00 | neg | 624 |  |  |  |
|  | SK 307 | 6 | 0.00 | 0.00 | 0.67 | neg | 446 | 120055 |  |  |
|  |  |  |  |  |  |  |  |  |  |  |
| **87** | SK 308 | Baseline | 0.67 | 0.33 | 0.33 | neg | 516 |  |  |  |
|  | SK 308 | 3 | 0.33 | 0.33 | 0.00 | neg | 527 | < 40.0 |  |  |
|  | SK 308 | 6 | 2.00 | 1.33 | 1.00 | neg | 394 |  |  |  |
|  |  |  |  |  |  |  |  |  |  |  |
| **88** | SK 316 | Baseline | 5.33 | 18.33 | 4.67 | pos (CFP10) | 461 | 5534 |  |  |
|  | SK 316 | 3 | 20.33 | 34.00 | 0.67 | pos (ESAT6/CFP10) | 539 |  |  |  |
|  | SK 316 | 6 | 3.00 | 15.33 | 0.67 | pos (CFP10) | 547 | 4039 |  |  |
|  | SK 316 | 9 | 0.00 | 0.00 | 0.00 | INDETERMINATE | 469 | 7342 |  |  |
|  |  |  |  |  |  |  |  |  |  |  |
| **89** | SK 317 | Baseline | 3.33 | 2.67 | 2.00 | neg | 545 |  |  |  |
|  | SK 317 | 3 | 0.67 | 1.33 | 0.33 | neg | 709 | 71 |  |  |
|  | SK 317 | 6 | 0.33 | 2.67 | 0.33 | neg | 689 |  |  |  |
|  | SK 317 | 9 | 2.00 | 3.33 | 2.67 | neg | 664 | 277 |  |  |
|  | SK 317 | 12 | 0.00 | 0.67 | 0.00 | neg | 747 |  |  |  |
|  |  |  |  |  |  |  |  |  |  |  |
| **90** | SK 318 | Baseline | 0.67 | 0.00 | 2.00 | neg | 295 |  |  |  |
|  | SK 318 | 3 | 0.33 | 0.00 | 0.00 | neg | 554 | 9086 |  |  |
|  | SK 318 | 6 | 4.67 | 0.00 | 0.00 | pos (ESAT6) | 372 |  |  |  |
|  | SK 318 | 9 | 0.00 | 0.00 | 0.33 | neg | 362 | 12122 |  |  |
|  | SK 318 | 12 | 0.00 | 0.00 | 0.00 | neg | 467 |  |  |  |
|  | SK 318 | 15 | 0.00 | 0.00 | 0.00 | INDETERMINATE | 402 | 21813 |  |  |
|  | SK 318 | 21 | 0.00 | 0.00 | 0.33 | neg | 369 |  |  |  |
|  |  |  |  |  |  |  |  |  |  |  |
| **91** | SK 320 | Baseline | 6.33 | 7.67 | 0.33 | pos (ESAT6/CFP10) | 901 | 2679 |  |  |
|  | SK 320 | 3 | 6.67 | 12.00 | 0.00 | pos (ESAT6/CFP10) | 780 |  |  |  |
|  | SK 320 | 6 | 9.33 | 17.67 | 0.33 | pos (ESAT6/CFP10) | 774 | 5914 |  |  |
|  | SK 320 | 9 | 3.00 | 14.67 | 0.00 | pos (CFP10) | 824 |  |  |  |
|  |  |  |  |  |  |  |  |  |  |  |
| **92** | SK 324 | Baseline | 6.67 | 16.00 | 0.33 | pos (ESAT6/CFP10) | 303 | 144252 |  |  |
|  | SK 324 | 3 | 2.00 | 4.33 | 1.33 | neg | 353 |  |  |  |
|  |  |  |  |  |  |  |  |  |  |  |
| **93** | SK 325 | Baseline | 1.33 | 2.00 | 0.33 | neg | 221 | 54285 |  |  |
|  | SK 325 | 3 | 1.00 | 0.33 | 0.00 | neg | 177 |  |  |  |
|  | SK 325 | 6 | 1.00 | 1.33 | 1.00 | neg | 164 | 75561 |  | Yes (treatment initiated 8 weeks previously) |
|  | SK 325 | 9 | 0.67 | 1.00 | 0.00 | neg | 268 |  | Yes | Yes |
|  | SK 325 | 12 | 0.00 | 0.00 | 0.67 | neg | 289 | 0 | Yes | Yes |
|  | SK 325 | 15 | 0.67 | 0.00 | 1.67 | neg | 302 |  | Yes |  |
|  | SK 325 | 21 | 64.33 | 60.67 | 0.00 | pos (ESAT6/CFP10) | 402 | <47 | Yes |  |
|  |  |  |  |  |  |  |  |  |  |  |
| **94** | SK 329 | Baseline | 8.00 | 18.67 | 1.33 | pos (ESAT6/CFP10) | 343 |  |  |  |
|  | SK 329 | 3 | 15.33 | 16.33 | 1.67 | pos (ESAT6/CFP10) | 545 | 3762 | Yes- on PMTCT |  |
|  | SK 329 | 6 | 4.67 | 7.67 | 1.67 | pos (CFP10) | 368 |  | Yes- on PMTCT |  |
|  | SK 329 | 9 | 9.33 | 27.67 | 2.00 | pos (ESAT6/CFP10) | 329 | 33553 | Yes- on PMTCT |  |
|  |  |  |  |  |  |  |  |  |  |  |
| **95** | SK 330 | Baseline | 38.00 | 36.00 | 2.00 | pos (ESAT6/CFP10) | 224 |  |  |  |
|  | SK 330 | 3 | 16.00 | 16.33 | 0.00 | pos (ESAT6/CFP10) | 238 | 26173 |  |  |
|  | SK 330 | 6 | 32.67 | 33.67 | 0.00 | pos (ESAT6/CFP10) | 253 |  |  |  |
|  | SK 330 | 9 | 0.67 | 1.00 | 0.33 | neg | 238 | 21702 |  |  |
|  | SK 330 | 12 | 22.33 | 16.33 | 18.00 | INDETERMINATE | 283 |  | Yes |  |
|  |  |  |  |  |  |  |  |  |  |  |
| **96** | SK 331 | Baseline | 84.67 | 1.00 | 0.33 | pos (ESAT6) | 223 |  |  |  |
|  | SK 331 | 3 | 0.67 | 1.33 | 0.33 | neg | 225 | 10180 |  |  |
|  | SK 331 | 6 | 0.33 | 0.67 | 0.00 | neg | 197 |  |  |  |
|  | SK 331 | 9 | 0.00 | 0.00 | 0.67 | neg | 306 | 313 | Yes |  |
|  | SK 331 | 12 | 0.00 | 0.67 | 0.33 | neg | 400 |  | Yes |  |
|  | SK 331 | 15 | 0.00 | 0.00 | 0.00 | neg | 290 | 6319 | Yes |  |
|  | SK 331 | 21 | 0.00 | 0.00 | 0.00 | neg | 456 |  | Yes |  |
|  |  |  |  |  |  |  |  |  |  |  |
| **97** | SK 334 | Baseline | 0.33 | 0.33 | 0.33 | neg | 123 |  |  |  |
|  | SK 334 | 3 | 1.33 | 2.00 | 0.00 | neg | 112 | 48240 |  |  |
|  | SK 334 | 6 | 2.00 | 2.67 | 1.67 | neg | 152 |  |  |  |
|  | SK 334 | 9 | 6.00 | 6.33 | 10.00 | neg | 112 | 31257 |  |  |
|  |  |  |  |  |  |  |  |  |  |  |
| **98** | SK 337 | Baseline | 0.00 | 0.00 | 0.33 | neg | 402 |  |  |  |
|  | SK 337 | 3 | 2.67 | 2.33 | 1.00 | neg | 343 | 1259 |  |  |
|  | SK 337 | 6 | 0.67 | 1.67 | 0.33 | neg | 367 |  |  |  |
|  | SK 337 | 9 | 1.67 | 2.67 | 0.67 | neg | 387 | 3799 |  |  |
|  | SK 337 | 12 | 0.00 | 0.33 | 0.33 | neg | 350 |  |  |  |
|  | SK 337 | 15 | 1.00 | 0.00 | 0.00 | neg | 291 | 3735 |  |  |
|  | SK 337 | 21 | 0.33 | 1.00 | 0.33 | neg | 392 |  |  |  |
|  |  |  |  |  |  |  |  |  |  |  |
| **99** | SK 338 | Baseline | 1.33 | 2.33 | 2.00 | neg | 165 | 66140 |  |  |
|  | SK 338 | 3 | 0.00 | 0.00 | 0.00 | neg | 252 |  |  |  |
|  | SK 338 | 6 | 2.33 | 2.00 | 2.00 | neg | 184 | 73251 |  |  |
|  | SK 338 | 9 | 3.67 | 2.33 | 1.33 | neg | 228 |  |  |  |
|  | SK 338 | 12 | 3.67 | 1.00 | 3.33 | neg | 404 | 489 | Yes |  |
|  |  |  |  |  |  |  |  |  |  |  |
| **100** | SK 341 | Baseline | 19.00 | 6.33 | 5.67 | pos (ESAT6) | 344 |  |  |  |
|  | SK 341 | 3 | 39.33 | 15.67 | 0.00 | pos (ESAT6/CFP10) | 299 | 525754 |  |  |
|  | SK 341 | 6 | 4.33 | 5.33 | 0.00 | pos (ESAT6/CFP10) | 642 |  | Yes |  |
|  | SK 341 | 9 | 27.33 | 8.67 | 4.00 | pos (ESAT6) | 769 | 169 | Yes |  |
|  |  |  |  |  |  |  |  |  |  |  |
| **101** | SK 342 | Baseline | 8.33 | 6.33 | 1.67 | pos (ESAT6/CFP10) | 415 |  |  |  |
|  | SK 342 | 3 | 5.00 | 3.33 | 1.33 | neg | 564 | < 40.0 |  |  |
|  | SK 342 | 6 | 5.67 | 2.00 | 1.33 | pos (ESAT6) | 400 |  |  |  |
|  | SK 342 | 9 | 1.67 | 2.00 | 0.33 | neg | 262 | 95 |  |  |
|  | SK 342 | 12 | 4.33 | 1.00 | 0.00 | pos (ESAT6) | 267 |  |  |  |
|  |  |  |  |  |  |  |  |  |  |  |
| **102** | SK 344 | Baseline | 5.33 | 27.33 | 0.00 | pos (ESAT6/CFP10) | 518 |  |  |  |
|  | SK 344 | 3 | 0.67 | 12.33 | 0.00 | pos (CFP10) | 416 | 2320 |  |  |
|  | SK 344 | 6 | 9.00 | 27.33 | 0.67 | pos (ESAT6/CFP10) | 363 |  |  |  |
|  | SK 344 | 9 | 0.67 | 35.00 | 1.33 | pos (CFP10) | 498 | 4032 |  |  |
|  |  |  |  |  |  |  |  |  |  |  |
| **103** | SK 348 | Baseline | 0.00 | 0.00 | 1.00 | neg | 536 | < 40.0 |  |  |
|  | SK 348 | 3 | 0.33 | 1.00 | 0.00 | neg | 678 |  |  |  |
|  | SK 348 | 6 | 1.00 | 0.33 | 1.33 | neg | 716 | 861 |  |  |
|  |  |  |  |  |  |  |  |  |  |  |
| **104** | SK 349 | Baseline | 1.33 | 0.67 | 0.00 | neg | 261 | 51064 |  |  |
|  | SK 349 | 3 | 2.33 | 1.67 | 2.00 | neg | 366 |  |  |  |
|  | SK 349 | 6 | 1.33 | 3.33 | 1.67 | neg | 230 | 19274 |  |  |
|  |  |  |  |  |  |  |  |  |  |  |
| **105** | SK 351 | Baseline | 37.67 | 91.67 | 5.33 | pos (ESAT6/CFP10) | 270 | 146024 |  |  |
|  | SK 351 | 3 | 19.33 | 4.00 | 0.00 | pos (ESAT6) | 351 |  |  | Yes |
|  | SK 351 | 6 | 9.00 | 6.00 | 0.33 | pos (ESAT6/CFP10) | 286 | 285891 |  | Yes |
|  | SK 351 | 9 | 8.33 | 1.67 | 1.33 | pos (ESAT6) | 196 |  |  |  |
|  | SK 351 | 12 | 19.00 | 1.00 | 0.00 | pos (ESAT6) | 279 | 1759 | Yes |  |
|  | SK 351 | 15 | 2.00 | 0.00 | 0.00 | neg | 282 |  | Yes |  |
|  |  |  |  |  |  |  |  |  |  |  |
| **106** | SK 353 | Baseline | 2.00 | 0.67 | 1.00 | neg | 474 |  |  |  |
|  | SK 353 | 3 | 1.33 | 1.33 | 1.33 | neg | 333 | 58237 |  |  |
|  | SK 353 | 6 | 5.67 | 3.67 | 3.00 | neg | 382 |  |  |  |
|  | SK 353 | 9 | 1.67 | 1.00 | 0.33 | neg | 423 |  |  |  |
|  | SK 353 | 12 | 0.00 | 0.00 | 0.00 | neg | 390 |  |  |  |
|  | SK 353 | 15 | 0.00 | 0.33 | 0.33 | neg | 352 | 807 |  |  |
|  |  |  |  |  |  |  |  |  |  |  |
| **107** | SK 354 | Baseline | 0.00 | 0.00 | 0.00 | INDETERMINATE | 180 |  |  |  |
|  | SK 354 | 3 | 4.33 | 9.67 | 0.33 | pos (CFP10) | 240 | 125 | Yes |  |
|  | SK 354 | 6 | 1.67 | 5.00 | 1.33 | neg | 269 |  | Yes |  |
|  | SK 354 | 9 | 1.67 | 3.33 | 0.00 | neg | 247 | 3614 | Yes |  |
|  |  |  |  |  |  |  |  |  |  |  |
| **108** | SK 357 | Baseline | 1.00 | 0.67 | 0.00 | neg | 237 |  |  |  |
|  | SK 357 | 3 | 12.00 | 12.67 | 0.33 | pos (ESAT6/CFP10) | 192 | 80582 |  |  |
|  | SK 357 | 6 | 10.33 | 4.67 | 1.00 | pos (ESAT6) | 195 |  |  |  |
|  | SK 357 | 9 | 36.00 | 12.67 | 0.00 | pos (ESAT6/CFP10) | 148 | 58024 |  |  |
|  | SK 357 | 12 | 0.00 | 0.00 | 0.00 | INDETERMINATE | 190 |  | Yes |  |
|  | SK 357 | 15 | 2.00 | 4.33 | 0.00 | pos (CFP10) | 320 | 60211 | Yes |  |
|  |  |  |  |  |  |  |  |  |  |  |
| **109** | SK 359 | Baseline | 23.33 | 5.33 | 0.67 | pos (ESAT6/CFP10) | 373 |  |  |  |
|  | SK 359 | 3 | 9.33 | 2.00 | 0.33 | pos (ESAT6) | 466 | 8711 |  |  |
|  | SK 359 | 6 | 32.00 | 2.33 | 0.00 | pos (ESAT6) | 426 |  |  |  |
|  | SK 359 | 9 | 5.67 | 0.33 | 0.00 | pos (ESAT6) | 357 | 19430 |  |  |
|  | SK 359 | 12 | 84.00 | 2.00 | 0.00 | pos (ESAT6) | 427 |  |  |  |
|  |  |  |  |  |  |  |  |  |  |  |
| **110** | SK 362 | Baseline | 1.00 | 0.67 | 0.67 | neg | 341 | 463 |  |  |
|  | SK 362 | 3 | 1.00 | 5.00 | 0.67 | pos (CFP10) | 572 |  |  |  |
|  | SK 362 | 6 | 2.33 | 2.33 | 0.00 | neg | 406 | 789 |  |  |
|  | SK 362 | 9 | 0.67 | 1.33 | 1.33 | neg | 448 |  |  |  |
|  | SK 362 | 12 | 0.67 | 2.67 | 0.00 | neg | 458 | 560 |  |  |
|  | SK 362 | 15 | 2.00 | 0.67 | 2.33 | neg | 510 |  |  |  |
|  | SK 362 | 21 | 0.67 | 2.33 | 0.33 | neg | 413 |  |  |  |
|  |  |  |  |  |  |  |  |  |  |  |
| **111** | SK 364 | Baseline | 1.00 | 34.33 | 1.00 | pos (CFP10) | 244 |  |  |  |
|  | SK 364 | 3 | 1.33 | 23.00 | 0.33 | pos (CFP10) | 285 | 879 |  |  |
|  | SK 364 | 6 | 0.67 | 107.67 | 0.00 | pos (CFP10) | 245 |  |  |  |
|  | SK 364 | 9 | 0.00 | 66.33 | 1.00 | pos (CFP10) | 222 | 1404 |  |  |
|  | SK 364 | 12 | 0.00 | 0.00 | 0.00 | INDETERMINATE | 370 |  |  |  |
|  | SK 364 | 15 | 1.33 | 1.33 | 0.33 | neg | 549 | 903 |  |  |
|  |  |  |  |  |  |  |  |  |  |  |
| **112** | SK 365 | Baseline | 0.67 | 1.00 | 1.33 | neg | 228 |  |  |  |
|  | SK 365 | 3 | 1.33 | 0.33 | 0.33 | neg | 229 | 32915 |  |  |
|  | SK 365 | 6 | 0.33 | 0.33 | 0.67 | neg | 200 |  |  |  |
|  | SK 365 | 9 | 0.00 | 0.00 | 0.00 | neg | 162 | 25979 |  |  |
|  | SK 365 | 12 | 20.33 | 17.67 | 14.00 | INDETERMINATE | 143 |  |  |  |
|  |  |  |  |  |  |  |  |  |  |  |
| **113** | SK 367 | Baseline | 0.33 | 1.67 | 0.33 | neg | 227 | 954 |  |  |
|  | SK 367 | 3 | 0.00 | 3.33 | 0.67 | neg | 249 |  |  |  |
|  | SK 367 | 6 | 1.00 | 1.67 | 1.00 | neg | 252 | 935 |  |  |
|  | SK 367 | 9 | 0.00 | 0.33 | 0.00 | neg | 296 |  |  |  |
|  |  |  |  |  |  |  |  |  |  |  |
| **114** | SK 368 | Baseline | 57.33 | 1.00 | 26.33 | INDETERMINATE | 484 | 14281 |  |  |
|  | SK 368 | 3 | 0.33 | 0.00 | 0.00 | neg | 422 |  |  |  |
|  |  |  |  |  |  |  |  |  |  |  |
| **115** | SK 370 | Baseline | 0.33 | 0.67 | 0.33 | neg | 191 | 1472 |  |  |
|  | SK 370 | 3 | 2.33 | 2.33 | 1.67 | neg | 233 |  |  |  |
|  | SK 370 | 6 | 0.33 | 0.67 | 0.33 | neg | 207 | 1232 |  |  |
|  | SK 370 | 9 | 3.33 | 4.67 | 1.67 | neg | 288 |  | Yes |  |
|  | SK 370 | 12 | BO | BO | BO | INDETERMINATE | 323 | 0 | Yes |  |
|  |  |  |  |  |  |  |  |  |  |  |
| **116** | SK 371 | Baseline | 1.33 | 0.33 | 1.33 | neg | 321 |  |  |  |
|  | SK 371 | 3 | 0.33 | 1.33 | 0.00 | neg | 298 |  |  |  |
|  | SK 371 | 6 | 0.33 | 3.00 | 0.67 | neg | 285 | 32807 |  |  |
|  | SK 371 | 9 | 0.67 | 3.67 | 0.33 | neg | 321 |  |  |  |
|  | SK 371 | 12 | 0.00 | 2.67 | 0.00 | neg | 246 | 144506 |  |  |
|  |  |  |  |  |  |  |  |  |  |  |
| **117** | SK 372 | Baseline | 0.33 | 2.00 | 0.33 | neg | 143 | 25290 |  |  |
|  | SK 372 | 3 | 10.00 | 4.00 | 0.33 | pos (ESAT6) | 106 |  |  |  |
|  | SK 372 | 6 | 4.33 | 2.67 | 0.33 | neg | 139 |  |  |  |
|  | SK 372 | 9 | 15.33 | 7.00 | 0.33 | pos (ESAT6/CFP10) | 139 | 12043 |  |  |
|  |  |  |  |  |  |  |  |  |  |  |
| **118** | SK 373 | Baseline | 0.33 | 0.67 | 0.00 | neg | 268 |  |  |  |
|  | SK 373 | 3 | 14.00 | 3.67 | 0.00 | pos (ESAT6) | 325 | 76397 |  |  |
|  | SK 373 | 6 | 9.67 | 4.00 | 0.00 | pos (ESAT6) | 261 |  |  |  |
|  | SK 373 | 9 | 3.00 | 0.67 | 0.33 | neg | 184 | 86734 |  |  |
|  | SK 373 | 12 | 28.67 | 8.00 | 0.33 | pos (ESAT6/CFP10) | 357 | <40 | Yes |  |
|  | SK 373 | 15 | 0.00 | 0.00 | 0.00 | neg | 584 | 517 | Yes |  |
|  | SK 373 | 21 | 0.00 | 0.00 | 0.00 | neg | 235 |  | Yes |  |
|  |  |  |  |  |  |  |  |  |  |  |
| **119** | SK 374 | Baseline | 7.00 | 5.00 | 6.00 | neg | 408 | 3586 |  |  |
|  | SK 374 | 3 | 0.00 | 0.33 | 0.00 | neg | 554 |  |  |  |
|  | SK 374 | 6 | 2.33 | 3.67 | 7.00 | neg | 549 |  |  |  |
|  | SK 374 | 9 | 2.33 | 1.67 | 0.00 | neg | 693 |  |  |  |
|  | SK 374 | 12 | 0.67 | 0.00 | 0.00 | neg | 581 | 1408 |  |  |
|  |  |  |  |  |  |  |  |  |  |  |
| **120** | SK 377 | Baseline | 5.67 | 8.67 | 0.67 | pos (ESAT6/CFP10) | 358 |  |  |  |
|  | SK 377 | 3 | 32.00 | 4.67 | 0.67 | pos (ESAT6) | 576 | 1025 |  |  |
|  | SK 377 | 6 | 3.00 | 9.33 | 3.67 | neg | 469 |  |  |  |
|  | SK 377 | 9 | 4.33 | 9.00 | 0.67 | pos (CFP10) | 489 | 29671 |  |  |
|  | SK 377 | 12 | 0.33 | 0.67 | 0.00 | neg | 483 |  |  |  |
|  | SK 377 | 15 | 23.67 | 8.00 | 4.67 | pos (ESAT6) | 359 |  |  |  |
|  |  |  |  |  |  |  |  |  |  |  |
| **121** | SK 378 | Baseline | 3.00 | 1.33 | 1.67 | neg | 544 | 2794 |  |  |
|  | SK 378 | 3 | 3.00 | 3.00 | 0.67 | neg | 443 |  |  |  |
|  | SK 378 | 6 | 12.00 | 2.33 | 0.33 | pos (ESAT6) | 502 | 5353 |  |  |
|  | SK 378 | 9 | 0.67 | 0.33 | 0.67 | neg | 554 |  |  |  |
|  | SK 378 | 12 | 14.00 | 3.67 | 0.00 | pos (ESAT6) | 486 | 6614 |  |  |
|  | SK 378 | 15 | 1.00 | 0.00 | 0.00 | neg | 432 |  |  |  |
|  | SK 378 | 21 | 1.67 | 0.33 | 0.00 | neg | 457 |  |  |  |
|  |  |  |  |  |  |  |  |  |  |  |
| **122** | SK 379 | Baseline | 14.67 | 12.33 | 4.67 | pos (ESAT6) | 199 |  |  |  |
|  | SK 379 | 3 | 4.00 | 2.00 | 0.67 | neg | 244 | 1785 |  |  |
|  | SK 379 | 6 | 0.00 | 4.67 | 0.00 | pos (CFP10) | 223 |  |  |  |
|  | SK 379 | 9 | 0.00 | 1.33 | 0.00 | neg | 247 | 2925 |  |  |
|  | SK 379 | 12 | 0.67 | 0.33 | 0.00 | INDETERMINATE | 165 |  |  |  |
|  |  |  |  |  |  |  |  |  |  |  |
| **123** | SK 381 | Baseline | 46.33 | 47.67 | 2.00 | pos (ESAT6/CFP10) | 198 |  |  |  |
|  | SK 381 | 3 | 10.33 | BO | 0.00 | pos (ESAT6/CFP10) | 276 | 382 | Yes |  |
|  | SK 381 | 6 | 21.33 | 49.67 | 1.67 | pos (ESAT6/CFP10) | 303 |  | Yes |  |
|  | SK 381 | 9 | 4.67 | 13.33 | 0.00 | pos (ESAT6/CFP10) | 227 | 177 | Yes |  |
|  |  |  |  |  |  |  |  |  |  |  |
| **124** | SK 382 | Baseline | 20.00 | 14.00 | 0.67 | pos (ESAT6/CFP10) | 390 | 98386 |  |  |
|  | SK 382 | 3 | 46.67 | 15.33 | 0.33 | pos (ESAT6/CFP10) | 427 | 101603 |  |  |
|  | SK 382 | 6 | 52.67 | 14.33 | 0.67 | neg | 235 |  |  |  |
|  | SK 382 | 9 | 45.33 | 18.00 | 36.67 | INDETERMINATE | 264 | 214669 |  |  |
|  |  |  |  |  |  |  |  |  |  |  |
| **125** | SK 383 | Baseline | 0.33 | 0.67 | 0.33 | neg | 273 | 29647 |  |  |
|  | SK 383 | 3 | 3.33 | 1.67 | 0.00 | neg | 280 |  |  |  |
|  | SK 383 | 6 | 0.00 | 0.00 | 0.00 | INDETERMINATE | 280 | 90228 |  |  |
|  | SK 383 | 9 | 0.00 | 0.00 | 0.00 | neg | 447 |  |  |  |
|  | SK 383 | 12 | 7.00 | 0.00 | 0.00 | pos (ESAT6) | 346 | 46171 |  |  |
|  |  |  |  |  |  |  |  |  |  |  |
| **126** | SK 384 | Baseline | 4.00 | 4.33 | 0.67 | neg | 519 | 8912 |  |  |
|  | SK 384 | 3 | 4.00 | 3.33 | 1.33 | neg | 495 |  |  |  |
|  | SK 384 | 6 | 1.00 | 2.33 | 0.00 | neg | 453 | 16065 |  |  |
|  | SK 384 | 9 | 16.33 | 12.00 | 0.33 | pos (ESAT6/CFP10) | 470 |  |  |  |
|  |  |  |  |  |  |  |  |  |  |  |
| **127** | SK 385 | Baseline | 2.00 | 2.00 | 0.33 | neg | 757 |  |  |  |
|  | SK 385 | 3 | 5.33 | 0.33 | 0.67 | pos (ESAT6) | 780 | 1276 |  |  |
|  | SK 385 | 6 | 2.00 | 0.33 | 1.33 | neg | 898 |  |  |  |
|  | SK 385 | 9 | 0.33 | 0.67 | 0.00 | neg | 877 | 1933 |  |  |
|  |  |  |  |  |  |  |  |  |  |  |
| **128** | SK 386 | Baseline | 16.00 | 3.33 | 4.00 | pos (ESAT6) | 409 | 6512 |  |  |
|  | SK 386 | 3 | 0.00 | 0.33 | 0.00 | neg | 357 | 27047 |  |  |
|  | SK 386 | 6 | 1.00 | 0.33 | 0.33 | neg | 357 |  |  |  |
|  | SK 386 | 9 | 1.00 | 0.67 | 0.00 | neg | 326 |  |  |  |
|  | SK 386 | 12 | 0.00 | 0.00 | 0.00 | neg | 384 | 35127 |  |  |
|  |  |  |  |  |  |  |  |  |  |  |
| **129** | SK 387 | Baseline | 11.33 | 5.33 | 1.67 | pos (ESAT6) | 184 |  |  |  |
|  | SK 387 | 3 | 5.33 | 1.67 | 1.00 | pos (ESAT6) | 379 | 2682 | Yes |  |
|  | SK 387 | 6 | 1.00 | 0.00 | 0.00 | neg | 381 |  | Yes |  |
|  | SK 387 | 9 | 1.33 | 0.00 | 0.00 | neg | 470 | 574 | Yes |  |
|  | SK 387 | 12 | 3.33 | 0.67 | 0.00 | neg | 408 |  | Yes |  |
|  |  |  |  |  |  |  |  |  |  |  |
| **130** | SK 388 | Baseline | 0.33 | 0.67 | 0.00 | neg | 364 |  |  |  |
|  | SK 388 | 3 | 1.00 | 1.33 | 0.67 | neg | 416 |  |  |  |
|  | SK 388 | 6 | 1.00 | 0.33 | 0.00 | neg | 298 |  |  |  |
|  | SK 388 | 9 | 0.67 | 0.33 | 0.00 | neg | 233 | 1276 |  |  |
|  |  |  |  |  |  |  |  |  |  |  |
| **131** | SK 389 | Baseline | 1.00 | 0.67 | 0.00 | neg | 677 | 419 |  |  |
|  | SK 389 | 3 | 17.67 | 7.00 | 0.00 | pos (ESAT6/CFP10) | 693 |  |  |  |
|  | SK 389 | 6 | 12.67 | 9.00 | 0.67 | pos (ESAT6/CFP10) | 734 | 792 |  |  |
|  | SK 389 | 9 | 8.00 | 1.33 | 1.00 | pos (ESAT6) | 548 |  |  |  |
|  | SK 389 | 12 | 65.67 | 21.33 | 11.67 | INDETERMINATE | 652 | 381 |  |  |
|  |  |  |  |  |  |  |  |  |  |  |
| **132** | SK 391 | Baseline | 68.00 | 7.00 | 0.67 | pos (ESAT6/CFP10) | 247 | 1936 |  |  |
|  | SK 391 | 3 | 6.00 | 11.67 | 3.33 | pos (CFP10) | 309 | 20767 |  |  |
|  | SK 391 | 6 | 4.00 | 2.00 | 1.33 | neg | 247 |  |  |  |
|  | SK 391 | 9 | 1.67 | 5.00 | 0.00 | pos (CFP10) | 280 | 13396 |  |  |
|  |  |  |  |  |  |  |  |  |  |  |
| **133** | SK 397 | Baseline | 5.67 | 0.00 | 0.00 | pos (ESAT6) | 332 |  |  |  |
|  | SK 397 | 3 | 0.33 | 0.00 | 0.33 | neg | 213 | 198266 |  |  |
|  | SK 397 | 6 | 6.67 | 3.33 | 0.00 | pos (ESAT6) | 190 |  |  |  |
|  | SK 397 | 9 | 4.33 | 0.33 | 0.67 | neg | 174 | 39194 |  |  |
|  |  |  |  |  |  |  |  |  |  |  |
| **134** | SK 398 | Baseline | 0.00 | 1.33 | 0.00 | neg | 225 |  |  |  |
|  | SK 398 | 3 | 0.33 | 0.00 | 0.00 | neg | 247 | 137533 |  |  |
|  | SK 398 | 6 | 0.67 | 0.33 | 0.33 | neg | 234 |  |  |  |
|  | SK 398 | 9 | 4.33 | 3.00 | 11.00 | INDETERMINATE | 248 | 4230 | Yes |  |
|  |  |  |  |  |  |  |  |  |  |  |
| **135** | SK 399 | Baseline | 0.33 | 3.00 | 0.00 | neg | 265 |  |  |  |
|  | SK 399 | 3 | 2.00 | 3.00 | 0.00 | neg | 220 |  |  |  |
|  | SK 399 | 6 | 1.33 | 0.67 | 0.33 | neg | 271 |  |  |  |
|  | SK 399 | 9 | 0.67 | 0.33 | 0.00 | neg | 242 | 3896 |  |  |
|  |  |  |  |  |  |  |  |  |  |  |
| **136** | SK 402 | Baseline | 31.00 | 33.67 | 1.00 | pos (ESAT6/CFP10) | 444 |  |  |  |
|  | SK 402 | 3 | 6.33 | 21.00 | 0.00 | pos (ESAT6/CFP10) | 502 | 113859 |  |  |
|  | SK 402 | 6 | 4.00 | 54.33 | 0.00 | pos (CFP10) | 542 |  |  |  |
|  | SK 402 | 9 | 5.33 | 22.33 | 0.00 | pos (ESAT6/CFP10) | 530 | 159184 |  |  |
|  |  |  |  |  |  |  |  |  |  |  |
| **137** | SK 404 | Baseline | 1.33 | 1.67 | 1.33 | neg | 430 | 12842 |  |  |
|  | SK 404 | 3 | 1.33 | 3.00 | 2.00 | neg | 512 |  |  |  |
|  | SK 404 | 6 | 3.67 | 1.33 | 5.67 | neg | 597 | 35375 |  |  |
|  | SK 404 | 9 | 0.33 | 0.33 | 0.00 | neg | 686 |  |  |  |
|  | SK 404 | 12 | 0.33 | 0.00 | 0.00 | neg | 497 | 35375 |  |  |
|  |  |  |  |  |  |  |  |  |  |  |
| **138** | SK 406 | Baseline | 2.00 | 5.00 | 0.67 | pos (CFP10) | 382 |  |  |  |
|  | SK 406 | 3 | 4.00 | 3.67 | 2.33 | neg | 328 | 35375 |  |  |
|  | SK 406 | 6 | 0.67 | 1.00 | 0.33 | neg | 347 |  |  |  |
|  | SK 406 | 9 | 1.33 | 0.33 | 0.00 | neg | 356 | 16284 |  |  |
|  | SK 406 | 12 | 20.00 | 27.00 | 0.33 | pos (ESAT6/CFP10) | 319 |  |  |  |
|  |  |  |  |  |  |  |  |  |  |  |
| **139** | SK 407 | Baseline | 13.67 | 45.00 | 1.33 | pos (ESAT6/CFP10) | 667 |  |  |  |
|  | SK 407 | 3 | 20.00 | 41.33 | 0.00 | pos (ESAT6/CFP10) | 502 | 896 |  |  |
|  | SK 407 | 6 | 0.00 | 0.00 | 0.00 | INDETERMINATE | 561 |  |  |  |
|  | SK 407 | 9 | 8.00 | 19.67 | 0.67 | pos (ESAT6/CFP10) | 595 | 1560 |  |  |
|  | SK 407 | 12 | 1.33 | 20.00 | 1.33 | pos (CFP10) | 616 |  |  |  |
|  | SK 407 | 15 | 4.67 | 9.00 | 0.00 | pos (ESAT6/CFP10) | 552 | 695 |  |  |
|  | SK 407 | 21 | 3.33 | 16.33 | 0.00 | pos (CFP10) | 577 |  |  |  |
|  |  |  |  |  |  |  |  |  |  |  |
| **140** | SK 409 | Baseline | 0.33 | 0.00 | 0.67 | neg | 322 |  |  |  |
|  | SK 409 | 3 | 0.00 | 0.33 | 0.00 | neg | 351 | 7468 |  |  |
|  | SK 409 | 6 | 0.00 | 0.00 | 0.00 | neg | 283 |  |  |  |
|  | SK 409 | 9 | 0.00 | 0.00 | 0.00 | INDETERMINATE | 317 | 17197 |  |  |
|  |  |  |  |  |  |  |  |  |  |  |
| **141** | SK 410 | Baseline | 0.67 | 0.00 | 0.00 | neg | 712 | 2570 |  |  |
|  | SK 410 | 3 | 3.33 | 0.00 | 0.00 | neg | 605 |  |  |  |
|  | SK 410 | 6 | 3.67 | 4.33 | 1.00 | neg | 606 | 1737 |  |  |
|  | SK 410 | 9 | 0.00 | 0.00 | 0.00 | INDETERMINATE | 687.97 |  |  |  |
|  | SK 410 | 12 | 0.00 | 0.00 | 0.00 | INDETERMINATE | 577 | 1594 |  |  |
|  | SK 410 | 15 | 4.67 | 4.00 | 0.33 | pos (ESAT6) | 619 |  |  |  |
|  |  |  |  |  |  |  |  |  |  |  |
| **142** | SK 411 | Baseline | 1.00 | 3.00 | 1.67 | neg | 182 | 15681 |  |  |
|  | SK 411 | 3 | 0.33 | 1.00 | 0.00 | neg | 237 |  |  |  |
|  | SK 411 | 6 | 0.33 | 0.00 | 0.00 | neg | 302 | 20520 |  |  |
|  | SK 411 | 9 | 0.33 | 1.00 | 0.00 | neg | 244 |  |  |  |
|  | SK 411 | 12 | BO | BO | BO | INDETERMINATE | 379 | 58253 |  |  |
|  |  |  |  |  |  |  |  |  |  |  |
| **143** | SK 412 | Baseline | 0.33 | 0.67 | 0.33 | neg | 233 | 12690 |  |  |
|  | SK 412 | 3 | 7.33 | 4.67 | 0.00 | pos (ESAT6/CFP10) | 182 |  |  |  |
|  | SK 412 | 6 | 3.33 | 7.33 | 0.33 | pos (CFP10) | 155 | 12790 |  |  |
|  | SK 412 | 9 | 5.00 | 6.00 | 0.67 | pos (ESAT6/CFP10) | 204 |  |  |  |
|  | SK 412 | 12 | 0.00 | 0.00 | 0.00 | INDETERMINATE | 196 | 23708 |  |  |
|  | SK 412 | 15 | 6.67 | 7.00 | 0.00 | pos (ESAT6/CFP10) | 306 |  | Yes |  |
|  |  |  |  |  |  |  |  |  |  |  |
| **144** | SK 417 | Baseline | 0.33 | 4.67 | 0.67 | neg | 330 |  |  |  |
|  | SK 417 | 3 | 0.00 | 1.67 | 0.00 | neg | 330 | 1018 |  |  |
|  | SK 417 | 6 | 0.33 | 1.33 | 0.67 | neg | 283 |  |  |  |
|  | SK 417 | 9 | 0.00 | 0.00 | 0.00 | INDETERMINATE | 338 | 5821 |  |  |
|  |  |  |  |  |  |  |  |  |  |  |
| **145** | SK 418 | Baseline | 0.33 | 0.00 | 0.00 | neg | 250 |  |  |  |
|  | SK 418 | 3 | 1.33 | 0.33 | 2.00 | neg | 266 | 17425 |  |  |
|  | SK 418 | 6 | 0.33 | 1.33 | 1.00 | neg | 369 |  |  |  |
|  | SK 418 | 9 | BO | BO | BO | INDETERMINATE | 187 | 159 | Yes |  |
|  |  |  |  |  |  |  |  |  |  |  |
| **146** | SK 420 | Baseline | 4.33 | 2.33 | 1.33 | neg | 457 |  |  |  |
|  | SK 420 | 3 | 4.33 | 18.00 | 73.33 | INDETERMINATE | 563 | 1793 |  |  |
|  | SK 420 | 6 | 2.67 | 2.33 | 0.00 | neg | 644 |  |  |  |
|  | SK 420 | 9 | 0.00 | 0.67 | 0.00 | neg | 474 | 1250 |  |  |
|  |  |  |  |  |  |  |  |  |  |  |
| **147** | SK 421 | Baseline | 0.00 | 1.67 | 0.00 | neg | 546 |  |  |  |
|  | SK 421 | 3 | 1.33 | 1.67 | 0.33 | neg | 594 | 7436 |  |  |
|  | SK 421 | 6 | 0.33 | 0.67 | 0.33 | neg | 378 |  |  |  |
|  | SK 421 | 9 | 1.00 | 0.33 | 0.00 | neg | 572 | 13356 |  |  |
|  |  |  |  |  |  |  |  |  |  |  |
| **148** | SK 422 | Baseline | 1.00 | 1.00 | 0.33 | neg | 268 | 330818 |  |  |
|  | SK 422 | 3 | 0.67 | 1.00 | 0.33 | neg | 175 |  |  |  |
|  | SK 422 | 6 | 1.00 | 0.67 | 0.33 | neg | 116 | 4034 | Yes |  |
|  | SK 422 | 9 | 0.67 | 0.33 | 0.67 | neg | 106 |  | Yes |  |
|  | SK 422 | 12 | 0.00 | 0.00 | 0.00 | neg | 146 | 199 | Yes |  |
|  |  |  |  |  |  |  |  |  |  |  |
| **149** | SK 425 | Baseline | 9.33 | 10.00 | 14.33 | INDETERMINATE | 192 | 101600 |  |  |
|  | SK 425 | 3 | 12.67 | 6.00 | 0.33 | pos (ESAT6/CFP10) | 245 |  |  |  |
|  | SK 425 | 6 | 10.00 | 1.00 | 0.00 | pos (ESAT6) | 217 | 269813 |  |  |
|  | SK 425 | 9 | 15.00 | 1.00 | 0.00 | pos (ESAT6) | 75 |  |  |  |
|  |  |  |  |  |  |  |  |  |  |  |
| **150** | SK 427 | Baseline | 3.00 | 5.33 | 1.67 | neg | 187 | 166208 |  |  |
|  | SK 427 | 3 | 30.33 | 36.00 | 0.33 | pos (ESAT6/CFP10) | 213 |  |  |  |
|  | SK 427 | 6 | 21.67 | BO | 0.33 | pos (ESAT6/CFP10) | 258 | 27236 |  |  |
|  | SK 427 | 9 | 13.33 | 57.33 | 0.00 | pos (ESAT6/CFP10) | 187 |  |  |  |
|  | SK 427 | 12 | 0.00 | 0.00 | 0.00 | INDETERMINATE | 173 | 16928 |  |  |
|  |  |  |  |  |  |  |  |  |  |  |
| **151** | SK 430 | Baseline | 30.00 | 4.00 | 0.00 | pos (ESAT6) | 301 |  |  |  |
|  | SK 430 | 3 | 8.00 | 10.67 | 3.33 | pos (CFP10) | 384 | 8141 |  |  |
|  | SK 430 | 6 | 0.00 | 0.33 | 0.00 | neg | 256 |  |  |  |
|  | SK 430 | 9 | 0.67 | 1.67 | 1.00 | neg | 495 | 31193 |  |  |
|  | SK 430 | 12 | 0.00 | 8.00 | 0.67 | pos (CFP10) | 286 |  |  |  |
|  |  |  |  |  |  |  |  |  |  |  |
| **152** | SK 431 | Baseline | 7.33 | 17.67 | 1.00 | pos (ESAT6/CFP10) | 891 | < 40.0 |  |  |
|  | SK 431 | 3 | 2.67 | 2.67 | 0.67 | neg | 1046 |  |  |  |
|  | SK 431 | 6 | 3.67 | 5.00 | 0.67 | pos (CFP10) | 1145 | 140 |  |  |
|  | SK 431 | 9 | 15.00 | 17.33 | 0.33 | pos (ESAT6/CFP10) | 1101 |  |  |  |
|  |  |  |  |  |  |  |  |  |  |  |
| **153** | SK 432 | Baseline | 16.67 | 15.67 | 2.00 | pos (ESAT6/CFP10) | 249 | 60868 |  |  |
|  | SK 432 | 3 | 15.00 | 5.33 | 0.00 | pos (ESAT6/CFP10) | 281 |  |  |  |
|  | SK 432 | 6 | 13.33 | 12.00 | 1.33 | pos (ESAT6/CFP10) | 242 | 171791 |  |  |
|  | SK 432 | 9 | 12.33 | 4.00 | 0.00 | pos (ESAT6) | 261 |  |  |  |
|  | SK 432 | 12 | 40.33 | 19.67 | 3.33 | pos (ESAT6/CFP10) | 143 | 209671 |  |  |
|  |  |  |  |  |  |  |  |  |  |  |
| **154** | SK 433 | Baseline | 49.33 | 26.67 | 4.00 | pos (ESAT6/CFP10) | 487 |  |  |  |
|  | SK 433 | 3 | 1.67 | 18.00 | 1.00 | pos (CFP10) | 524 | 5461 |  |  |
|  | SK 433 | 6 | 0.00 | 0.00 | 0.00 | INDETERMINATE | 397 | 6444 |  |  |
|  |  |  |  |  |  |  |  |  |  |  |
| **155** | SK 434 | Baseline | 4.00 | 3.33 | 1.33 | neg | 403 |  |  |  |
|  | SK 434 | 3 | 19.33 | 11.33 | 0.00 | pos (ESAT6/CFP10) | 399 | 5421 |  |  |
|  | SK 434 | 6 | BO | 26.67 | 2.33 | pos (ESAT6/CFP10) | 597 |  |  |  |
|  | SK 434 | 9 | 32.00 | 0.67 | 0.00 | pos (ESAT6) | 286 | 5418 |  |  |
|  |  |  |  |  |  |  |  |  |  |  |
| **156** | SK 437 | Baseline | 0.67 | 1.00 | 0.67 | neg | 437 | 213518 |  |  |
|  | SK 437 | 3 | 0.67 | 0.33 | 0.00 | neg | 329 |  |  |  |
|  | SK 437 | 6 | 13.00 | 9.67 | 19.00 | INDETERMINATE | 425 | 526287 |  |  |
|  |  |  |  |  |  |  |  |  |  |  |
| **157** | SK 438 | Baseline | 10.33 | 35.67 | 2.33 | pos (ESAT6/CFP10) | 233 |  |  |  |
|  | SK 438 | 3 | 100.00 | 40.67 | 6.67 | pos (ESAT6/CFP10) | 212 | 116046 |  |  |
|  | SK 438 | 6 | BO | BO | 4.33 | pos (ESAT6/CFP10) | 205 | 285304 |  |  |
|  | SK 438 | 9 | 0.00 | 0.00 | 0.00 | INDETERMINATE | 128 |  |  |  |
|  |  |  |  |  |  |  |  |  |  |  |
| **158** | SK 440 | Baseline | 6.00 | 4.33 | 1.33 | pos (ESAT6) | 512 | 16787 |  |  |
|  | SK 440 | 3 | 5.00 | 2.67 | 0.00 | pos (ESAT6) | 409 |  |  |  |
|  |  |  |  |  |  |  |  |  |  |  |
| **159** | SK 444 | Baseline | 1.00 | 0.67 | 1.33 | neg | 293 | 40563 |  |  |
|  | SK 444 | 3 | 2.33 | 0.00 | 0.33 | neg | 290 |  |  |  |
|  | SK 444 | 6 | 0.33 | 0.67 | 1.33 | neg | 321 | 21447 |  |  |
|  | SK 444 | 9 | 0.67 | 1.33 | 1.00 | neg | 342 |  |  |  |
|  | SK 444 | 12 | 1.00 | 1.33 | 0.00 | neg | 298 | 13816 |  |  |
|  | SK 444 | 15 | 3.67 | 2.00 | 0.00 | INDETERMINATE | 215 |  |  |  |
|  | SK 444 | 21 | 2.67 | 6.00 | 0.33 | pos (CFP10) | 280 | 11267 |  |  |
|  |  |  |  |  |  |  |  |  |  |  |
| **160** | SK 447 | Baseline | 0.00 | 0.00 | 0.00 | neg | 582 | 2584 |  |  |
|  | SK 447 | 3 | 0.00 | 1.00 | 1.00 | neg | 639 |  |  |  |
|  | SK 447 | 6 | 1.33 | 1.00 | 0.00 | neg | 531 | 13329 |  |  |
|  | SK 447 | 9 | 0.33 | 0.00 | 0.33 | neg | 560 |  |  |  |
|  |  |  |  |  |  |  |  |  |  |  |
| **161** | SK 448 | Baseline | 1.33 | 1.33 | 0.33 | neg | 422 |  |  |  |
|  | SK 448 | 3 | 13.00 | 8.67 | 0.00 | pos (ESAT6/CFP10) | 441 | 27949 |  |  |
|  | SK 448 | 6 | 9.00 | 2.33 | 0.00 | pos (ESAT6) | 425 |  |  |  |
|  | SK 448 | 9 | 20.67 | 32.67 | 24.67 | INDETERMINATE | 386.65 | 22408 |  |  |
|  | SK 448 | 12 | 4.00 | 0.33 | 0.00 | neg | 403 |  |  |  |
|  |  |  |  |  |  |  |  |  |  |  |
| **162** | SK 449 | Baseline | 0.00 | 1.67 | 0.00 | neg | 336 | 6820 |  |  |
|  | SK 449 | 3 | 1.33 | 1.67 | 2.00 | neg | 316 |  |  |  |
|  | SK 449 | 6 | 0.00 | 1.00 | 0.33 | neg | 280 | 5619 |  |  |
|  | SK 449 | 9 | 0.00 | 0.33 | 0.00 | neg | 317 |  |  |  |
|  |  |  |  |  |  |  |  |  |  |  |
| **163** | SK 450 | Baseline | 0.00 | 0.00 | 0.00 | neg | 540 | 6609 |  |  |
|  | SK 450 | 3 | 0.00 | 0.00 | 0.00 | neg | 673 |  |  |  |
|  | SK 450 | 6 | 0.00 | 0.00 | 0.00 | neg | 572 | 2243 |  |  |
|  | SK 450 | 9 | 0.00 | 0.00 | 0.00 | neg | 540 |  |  |  |

**Supplementary Table 1: Longitudinal Elispot data for 163 study participants– BO indicates a ‘black out’ (Elispot well is purple and Spot Forming Cells can’t be individually counted).**
